# Assessing the Mechanism of Action of Synthetic Nanoengineered Antimicrobial Polymers against the Bacterial Membrane of *Pseudomonas aeruginosa*

**DOI:** 10.1021/acs.biomac.5c01175

**Published:** 2025-09-22

**Authors:** Ramón Garcia Maset, Laia Pasquina-Lemonche, Alexia Hapeshi, Luke A. Clifton, Jamie K. Hobbs, Freya Harrison, Sébastien Perrier, Stephen C. L. Hall

**Affiliations:** † Warwick Medical School, University of Warwick, Coventry CV4 7AL, U.K.; ‡ School of Biosciences, 7315 University of Sheffield, Sheffield S10 2TN, U.K.; § Department of Chemistry, University of Warwick, Coventry CV4 7AL, U.K.; ∥ ISIS Neutron and Muon Source, 97008 Rutherford Appleton Laboratory, Didcot OX11 0DE, U.K.; ⊥ School of Life Sciences, University of Warwick, Coventry CV4 7AL, U.K.; # School of Mathematical and Physical Sciences, University of Sheffield, Sheffield S3 7RH, U.K.; ¶ Faculty of Pharmacy and Pharmaceutical Sciences, Monash University, Parkville, Victoria 3052, Australia

## Abstract

The lack of appropriate antimicrobials to tackle multidrug-resistant Gram-negative bacteria poses an escalating threat to modern medicine. Addressing this urgent issue, we have recently developed synthetic nanoengineered antimicrobial polymers (SNAPs), inspired by the physicochemical properties of antimicrobial peptides. Our findings have demonstrated that SNAPs are potent antimicrobial agents characterized by low toxicity and cost-effective large-scale production. In this study, we elucidate the mechanism of action of two distinct SNAPs, which vary in length and charge distribution. Focusing on the Gram-negative pathogen *Pseudomonas aeruginosa* LESB58, a hypervirulent strain prevalent in cystic fibrosis patients, we employ advanced high-resolution imaging techniques and neutron reflectometry to uncover the precise interactions between SNAPs and the bacterial cell envelope. Our research identifies lipopolysaccharide as a critical target, detailing architecture-specific envelope disruptions, such as asymmetry loss, pore formation, and membrane dissolution. These insights into the structure–function relationships of SNAPs pave the way for the rational design of tailored antimicrobial polymers with specific targeted mechanisms of action.

## Graphical abstract

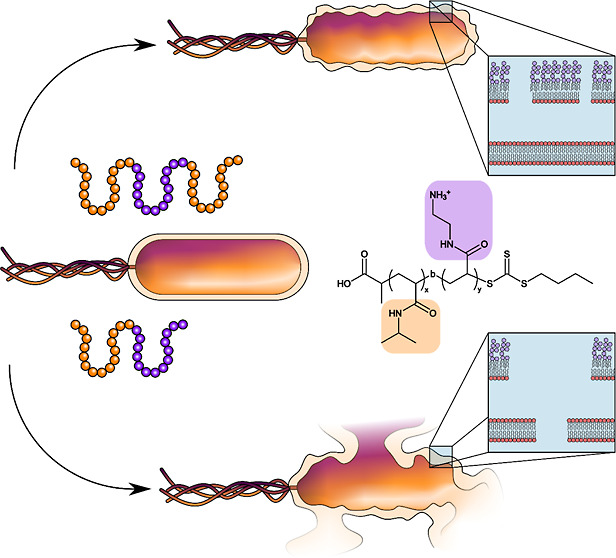

## Introduction

The spread of bacterial antimicrobial resistance (AMR) is a current and evolving threat to global health. It has been reported that AMR-related infections were involved in 4.95 million deaths in 2019, and a direct contributing factor of an estimated 1.27 million of these.^1^ Concerningly, the number of AMR-attributable deaths is expected to rise greatly to 10 million per annum by 2050,^2^ and this estimate was made even before the increase in AMR prevalence seen as a result of increased antibiotic use in the COVID-19 pandemic. While the drivers behind increasing AMR are many and complex,^3^ beyond the scope of this article, a key factor is the overuse of existing antimicrobials, which leads to increasing resistance, which is in turn partially symptomatic of the lack of effective new treatments.^4^ While progress developing new antimicrobials has not been wholly devoid, most of these have been effective in treating Gram-positive infections.^3,5^ In contrast, Gram-negative infections are notoriously difficult to treat. Their intrinsic resistance stems largely from the structure of their cell envelope, which severely limits the uptake and efficacy of many antibiotics, allowing resistance to be more easily acquired and resulting in the emergence of multidrug-resistant (MDR) strains.^3,6–10^ Central to this defense is the outer membrane (OM)—a highly asymmetric lipid bilayer composed of an outer leaflet of anionic lipopolysaccharides, stabilized by divalent cations, and an inner leaflet of glycerophospholipids. This unique architecture forms a potent physicochemical barrier to both hydrophilic and hydrophobic molecules, obstructing a broad spectrum of antimicrobial compounds from entry into the cell.^11^ Beyond passive impermeability, Gram-negative pathogens are further equipped with efficient broad-spectrum efflux pumps that actively expel antimicrobials from the cell.^11^ While the development of new antimicrobials has increasingly targeted efflux mechanisms, cell envelope components, and intracellular targets, these approaches often rely on specific molecular interactions.^12^ Single-target strategies are unfortunately particularly vulnerable to resistance, as even minor mutations in the target can render a drug ineffective.^12^ Conversely, antimicrobials with multiple or nonspecific targets offer a strategic advantage as they present fewer opportunities for bacteria to acquire resistance through isolated stochastic selective mutations.

As a possible strategy to combat rising resistance to traditional small-molecule antimicrobials, recent attention has turned to antimicrobial peptides (AMPs). AMPs are short peptides characterized by the presence of cationic amino acid residues, typically arginine or lysine, paired with hydrophobic residues.^13^ These structural features enable them to exert broad-spectrum antimicrobial activity, including against MDR pathogens. While there are exceptions, AMPs have been shown to have multiple, often simultaneous, mechanisms of action against Gram-negative bacteria. It is these multiple mechanisms of action that make resistance less likely to be acquired through stochastic mutation. Where resistance is acquired, the resistance adaptation evolves at a much lower rate in comparison with small-molecule antibiotics typically with a single target.^14^ In the case of colistin, for example, almost 50 years were needed before the spread of resistance.^15^ The most common mechanisms involve the electrostatic interaction between the cationic peptide and the anionic bacterial membrane, driving membrane binding and subsequent insertion of hydrophobic residues, ultimately leading to the disruption of membrane integrity.^13,16^ Polymyxins, for example, are a class of cyclic cationic peptides utilized as a last-resort antibiotic. While there remains some uncertainty as to the exact mechanism of action, it is well established that they target the Gram-negative cell envelope by binding and disrupting lipopolysaccharides (LPSs).^17,18^ Though LPS is a frequent target, the overarching specificity of AMPs for bacterial membranes is provided by the net negative charge of the bacterial cell surface contributed by anionic phospholipids such as phosphatidylglycerols and cardiolipin as well as anionic glycolipids (LPS in the case of Gram-negative bacteria, and lipoteichoic acids in the case of Gram-positive bacteria), all of which have been shown to play a role in AMP binding.^19^ While cell envelope disruption is a common feature among AMPs,^13,20,21^ it is often accompanied by other complementary modes of action. The human peptide LL-37, for example, has been shown to directly interact with bacterial membranes while also modulating immune responses to further enhance its antimicrobial activity.^13,20,22^ Furthermore, it has been demonstrated that AMPs can target intracellular nucleic acids, inhibiting transcription and translation processes to impart their bactericidal efficacy.^23^ Despite the promising activity of AMPs, their clinical adoption has been hindered by high cytotoxicity, narrow therapeutic indexes, poor bioavailability, and high production costs.^24,25^

As an alternative to AMPs, synthetic antimicrobial polymers (SNAPs) have emerged in recent years.^26^ The ease of synthetic polymer synthesis has potential to drastically reduce manufacturing costs, and advances in polymer chemistry enable the synthesis of well-defined polymers with fine control over key molecular parameters, including composition, molecular weight, block copolymer segmentation,^27–29^ and polymer architecture (e.g., linear polymers, stars, brushes, nanoparticles)^30–35^ while allowing functionalization with diverse chemical moieties. As such, polymer chemistry enables us to mimic the physicochemical properties of amino acid residues commonly found in AMPs,^36^ particularly cationic and hydrophobic groups, while allowing precise control over molecular design, granting synthetically straightforward fine-tuning of molecular properties to maximize the bactericidal efficacy and decrease the cytotoxicity against the patient’s cells.

Our work focuses on SNAPs containing *N*-isopropylacrylamide (NIPAM) and *N*-(2-aminoethyl) acrylamide (AEAM) as the hydrophobic and cationic constituents, mimicking the side chains of leucine and lysine residues, respectively, frequently found in AMPs. AEAM-containing polymers have been previously shown to be positively charged at physiological pH with the p*K*_a_ for the primary amine between 8 and 11, similar to the primary amine on lysine, which they are mimicking.^27^ These cationic, amphiphilic polymers have demonstrated selectivity toward negatively charged bacterial membranes, disruption of which leads to their antimicrobial activity. It has been identified that copolymers with segregation of these NIPAM and AEAM units in blocks show potent antimicrobial behavior, including against Gram-negative bacteria, more so than their statistical counterparts.^27^ An advantage of SNAPs is the ability to access complex molecular architectures to boost their efficacy. For example, linear copolymers of AEAM and NIPAM have been compared with their analogous star,^30,32^ or bottlebrush architectures.^34,37–39^ Despite the potential for improved performance with increased architectural complexity, in order to extract their maximum potential, it is vital to understand how the molecular design of the linear copolymer component of the SNAPs influences their mechanism of action and what effect this has in turn on their efficacy in different scenarios.

Previously, we have investigated the antimicrobial efficacy and biocompatibility of linear di- and triblock copolymers of AEAM and NIPAM, which differ in their molecular weight and block segmentation (a-D50 and a-T100-1, Figure 1).^40^ While both polymers exhibit antimicrobial activity against *Pseudomonas aeruginosa*, there are strain and media-specific differences in their efficacy, implying variations in their mechanism of action.^40^ Here, we investigate the specific mechanisms through which a-D50 and a-T100 disrupt the cellular envelope of *P. aeruginosa* LESB58, a clinical isolate from cystic fibrosis patients.^41^ This strain exemplifies the challenges in treating Gram-negative infections. Adaptation to the lung environment has driven the emergence of clinically relevant phenotypes, such as biofilm hyperproduction and increased resistance to common classes of antibiotics such as β-lactams, aminoglycosides, and quinolones, while simultaneously possessing a high number of genes in which mutations are frequently found in MDR-resistant bacteria, increasing the likelihood of the strain acquiring resistance to any given antibiotic.^42,43^ Like other Gram-negative bacteria, LESB58 possesses an OM enriched with LPS. However, genome sequencing indicates it likely expresses a rough phenotype, lacking the O-antigen and retaining only the core oligosaccharide.^43^ To elucidate the molecular mechanisms underlying SNAP bactericidal activity, we employed a combination of cellular imaging and biophysical analyses, identifying architecture-specific interactions with the bacterial cell envelope. Electron microscopy (EM) and atomic force microscopy (AFM) were used to visualize the effects of the copolymers on bacterial morphology. Fluorescence-based assays were employed to confirm polymer–LPS interactions in vitro. Finally, to gain molecular-level structural insight, we used neutron reflectometry (NR) to examine the interaction of SNAP with a biomimetic floating asymmetric membrane designed to mimic the OM of *P. aeruginosa* LESB58. These findings provide mechanistic insight into how SNAP architecture governs antimicrobial efficacy, informing future design strategies for polymer-based therapeutics targeting MDR Gram-negative pathogens.

**Figure 1:**
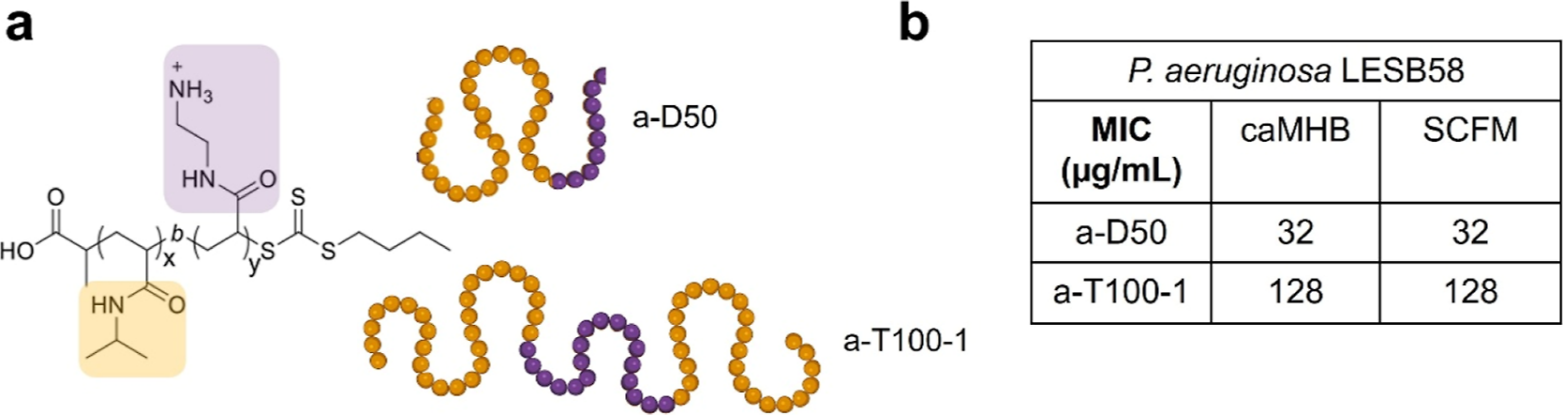
(a) Schematic representation of the antimicrobial polymers investigated throughout this study. Golden color indicates NIPAM blocks, and purple color indicates AEAM blocks. (b) Minimum Inhibitory Concentration (MIC) values expressed in μg/mL of the copolymers tested in Cation-adjusted Mueller Hinton Broth (caMHB) and synthetic cystic fibrosis medium (SCFM) against *P. aeruginosa* LESB58.

## Experimental Section

### Bacterial Strains and Culture Conditions

The Liverpool epidemic strain *P. aeruginosa* LESB58 was grown from cryovials in LB plates. A single colony from the plate was inoculated into 5 mL of caMHB and incubated at 37 °C with shaking for an overnight. Subsequently, the overnight culture was used to prepare a fresh inoculum in caMHB (OD_600_ = 0.1) and incubated at 37 °C with shaking until the midexponential phase was obtained (around 3 h).

### Scanning Electron Microscopy

From an overnight of bacterial suspension in caMHB, a fresh inoculum was prepared and incubated at 37 °C with shaking until the midexponential phase was obtained (10^7–8^ cfu/mL in caMHB). This bacterial solution was incubated in the presence of polymeric compounds (at the MIC and 2 × MIC) at 37 °C for 1 h. Then, the cells were pelleted by centrifugation at 6000*g* for 1 min, followed by 3 washes in PBS. 12 mm diameter circular glass coverslips were incubated with 50 μL of polylysine in a 24-well tissue culture plate. After 15 min, the polylysine solution was removed, and the coverslips were left to dry. The bacterial cell pellets were resuspended in 400 μL of PBS, and 100 μL was added to the coverslips. After 30 min of incubation, the excess volume was removed. The cells were then fixed at 4 °C overnight with a 2.5% glutaraldehyde solution in PBS. After fixation, the 2.5% glutaraldehyde solution was discarded, and the coverslips where rinsed 3 times with PBS. The coverslips were transferred to clean wells, and dehydration was performed using an ethanol gradient (from 20%, 50%, 70%, 90%, 100%, and 100%) for 10 min at each concentration. After complete dehydration, the coverslips were moved to clean wells and were incubated with 0.5 mL of hexamethyldisilazane (HDMS) as a drying agent for 30 min. The HDMS solution was then discarded, and the coverslips were moved to clean wells and left to dry in a laminar flow cabinet for 30 min. Copper tape was added to SEM sample holders, and the coverslips were placed on top. Finally, the samples were sputtered using a carbon coater (Emitech K950X). Imaging was performed at the Warwick Electron Microscopy Research Technology Platform on a Zeiss Gemini Scanning Electron Microscope equipped with an InLens detector at a voltage of 1 kV.

### Transmission Electron Microscopy

From an overnight of bacterial culture in caMHB, a fresh inoculum was prepared and incubated at 37 °C with shaking until the midexponential phase was obtained (∼10^8^ cfu/mL in caMHB). This bacterial solution was incubated in the presence of polymeric compounds at the MIC, at 37 °C for 1 h. Then, the cells were pelleted by centrifugation at 6000*g* for 1 min, followed by 3 washes in PBS. The cells were then fixed at 1 h at room temperature with a 2.5% glutaraldehyde solution in PBS. After fixation, the 2.5% glutaraldehyde solution was discarded, and the pellets were rinsed 3 times with PBS. Cells were subsequently incubated with 1% osmium tetroxide for 60 min at room temperature. Following washing with PBS, the bacterial samples were dehydrated in a graded acetone series and transferred to graded acetone: epoxy resin mixtures for 45 min each until pure resin incubated overnight at a constant temperature. Finally, the specimens were sectioned with an ultramicrotome (RMC). Imaging was performed at the Warwick Advanced Bioimaging Research Technology Platform on a Jeol 2100Plus LaB6 transmission electron microscope equipped with a Gatan OneView IS camera.

### Atomic Force Microscopy

The cells were grown following the same methodology as for scanning electron microscopy up until the point of 30 min incubation of the cell suspension on top of the polylysine-coated glass coverslip. Then, the excess volume was removed, and the cells were directly fixed with 2.5% glutaraldehyde solution in PBS for 1 h without drying. This fixation step was necessary; otherwise, no other method of surface adhesion was successful (after numerous attempts) with live *P. aeruginosa* LESB58 cells, probably due to the clinical nature of the strain. After fixation, the 2.5% glutaraldehyde solution was discarded, and the coverslips where rinsed 3 times with 1 mL of PBS. The cells were kept on top of the glass coverslip inside a 24-well plate in PBS overnight at RT. The next day, the excess PBS was removed, the bottom part of the glass was thoroughly dried (without affecting the sample) and glued to a rectangular microscopy glass slide and fixed to the AFM stage with ReproRuber green glue. 250 μL of PBS was added to the top of the sample, and liquid AFM was performed. The imaging was performed with a FastScan-D probe (Bruker FastScan Bio, Santa Barbara, CA, USA) calibrated using the Sader method: spring constant (*k*) = 0.117 N/m and sensitivity (*S*) = 30.12, using a Bruker FastScan Dimensions machine and Peak Force Tapping mode. The Peak Force frequency was kept at 2 kHz, the Peak Force Amplitude was kept between 80 and 150 nm, and the Peak Force set-point was kept between 0.5 and 2 nN. The integral gain oscillated between 1 and 8. All these parameters were optimized in situ to obtain the best tracking possible where the trace and retrace curves match as much as possible, indicating good cell adhesion.

### Lipopolysaccharide Binding Assay

The affinities of the interaction of polymeric materials to LPS were determined in a displacement assay by using the BODIPY-TR-cadaverine dye by slightly modifying the protocol previously described by Ouberai et al.^67^ The assay is based on the binding of LPS with the dye, which results in the quenching of the fluorescence signal. The displacement of the dye by the tested materials leads to the dequenching of the fluorescent signal of the BODIPY-TR-cadaverine dye. Stock solution of BODIPY-TR-cadaverine dye (10 μg/mL) and LPS from *P. aeruginosa* LESB58 (100 μg/mL) were prepared in Tris Buffer (pH 7.4, 50 mM). Polymers were dissolved in Tris Buffer (pH 7.4, 50 mM) at the desired concentration, and 50 μL of polymer solution was added to microwells followed by serial dilution. Then, in each well, 50 μL of the mixture of LPS: the BODIPY-TR-cadaverine dye (50/50% v/v) was added. The final concentration of LPS was 25 μg/mL and the final concentration of BODIPY-TR-cadaverine was 2.5 μg/mL. Each well was carefully mixed. After a short period of incubation in the dark, the fluorescence was determined (excitation wavelength, 580 nm; emission wavelength, 620 nm) by using a fluorescence spectrophotometer. Tris-buffer without polymeric material was used as a negative control. The experiment was performed in triplicate.

### Floating Asymmetric Bilayer Preparation

Silicon substrates (80 × 50 × 15 mm with a single 80 × 50 mm face polished to ∼3 Å rms roughness) were purchased from Crystran (Poole, UK) and subsequently coated in Permalloy (80:20 Fe/Ni) and Gold films of approximately 130 and 175 Å thickness, respectively, by the Nanofabrication Facility (National Institute of Standards and Technology, USA). Gold-coated substrates were cleaned via UV-Ozone treatment for 30 min, prior to extensive washing with ultrapure water and drying under a stream of N_2_. Cleaned substrates were functionalized with *N*-(2-hydroxyethyl)-16-mercaptohexadecanamide by immersion in a 60 μM solution prepared in ethanol for 48 h.

A clean, purpose-built Langmuir trough with a custom dipping arm and autolevelling device (Brown-Waite Engineering, Coventry, UK) was filled with a solution of 5 mM CaCl_2_ cooled to 10 °C. The SAM/Au/Permalloy-coated Si substrates were submerged into the subphase and a monolayer of tail-deuterated 1,2-dipalmitoyl-^2^H_62_-*sn*-glycero-3-phosphocholine (*d*_62_-DPPC, Avanti Polar Lipids, AL, USA) was spread at the air/waiter interface from a 2 mg/mL chloroform solution. After evaporation of the residual organic solvent, the monolayer was annealed through three cycles of compression to 38 mN m^–1^ and expansion to 0 mN m^–1^ until finally being compressed to 36 mN m^–1^ and maintained at this surface pressure. A Langmuir–Blodgett deposition of the inner leaflet was performed under a constant surface pressure of 36 mN m^–1^ and the polished face of the substrate being raised perpendicular to the air–water interface at a rate of 2 mm min^–1^. The trough was subsequently cleaned, and the subphase was replaced with a 5 mM CaCl_2_ solution, cooled to 10 °C. A monolayer of lipopolysaccharide from *Escherichia coli* EH100 Ra mutant (RaLPS) (Sigma-Aldrich, UK) was spread from a 2 mg/mL solution of 3:2 CHCl_3_/MeOH. After solvent evaporation, the RaLPS monolayer was annealed through three successive compression–expansion cycles from 38 mN m^–1^ and 0 mN m^–1^ and then held at a surface pressure of 36 mN m^–1^. The substrate was mounted on the dipping arm with the polished face of the substrate aligned to be parallel to the air/waiter interface. Langmuir–Schaeffer deposition was performed with the substrate lowered through the air/water interface at 2 mm min^–1^. Once fully submerged, the bilayer-deposited substrate was assembled into a solid–liquid flow cell for NR measurements.

### Neutron Reflectometry Data Collection

Neutron Reflectometry (NR) measures the elastic, specular reflection of neutrons as a function of momentum transfer perpendicular to the interface, *Q*_*z*_, as defined in eq 1

1 $$Q_{z}=\frac{4\pi \sin\theta }{\lambda }$$

where θ is the incident angle and λ is the neutron wavelength. The reflected intensity at a given value of *Q*_*z*_ is dependent on the structure and scattering length density (SLD) profile across the interface, where the SLD is defined as the sum of the coherent scattering lengths, *b*_c_, for each atomic nucleus, *n*, within a given molecular volume, *V*_m_. When the precise molecular volume of a component is unknown, the SLD can also be described in terms of mass density, ρ_m_, molecular weight, *M*_w_, and Avogadro’s constant, *N*_A_, as described in eq 2

2 $$SLD=\frac{\sum_{i=1}^{N}b_{c}}{V_{m}}=\frac{\rho_{m}N_{A}\sum_{i=1}^{N}b_{c}}{\sum_{i=1}^{N}M_{w}}$$

Neutrons display particular sensitivity to the difference in SLD between hydrogenous and deuterated material. By including differential isotopic labels within the sample, combined with varying the isotopic composition of the bulk solvent, so-called isotopic contrasts, the resultant reflected intensity will be sensitive to specific regions of the interfacial structure. In this study, we have exploited isotopic contrast through differential isotopic labeling of each leaflet within the floating asymmetric bilayer and variation of the SLD of the bulk solvent. For a more detailed description of NR, we direct the reader to the following refs 48 and 50 .

Neutron reflectometry (NR) was performed using the OFFSPEC reflectometer at the ISIS Neutron and Muon Source (STFC, Rutherford Appleton Laboratory, UK).^68^ Solid–liquid flow cells were mounted to kinematic mounts in a horizontal geometry. Aluminum top plates of the flow cells were connected to a recirculating water bath to maintain the sample temperature at 37 °C. PEEK base plates of the flow cell were connected to an HPLC pump for bulk isotopic contrast changes. Time-of-flight NR was measured at two incident angles: 0.7° and 2.0° using an incident wavelength range of 1.5–14 Å, covering an effective *Q*_*z*_ range of 0.012–0.3 Å^–1^ where δ*Q*_*z*_/*Q*_*z*_ = 5%.

The structure of the pristine bilayers was measured by NR in 20 mM HEPES, 2 mM CaCl_2_, pH 7.4, in four solution contrasts: D_2_O, H_2_O, gold-matched water (AuMW, 75% v/v D_2_O), and silicon-matched water (SiMW, 38% v/v D_2_O). Bulk solution contrasts were changed via a HPLC pump operating at a flow rate of 1 mL min^–1^. Polymers were prepared at a concentration of 0.5 μM in the same buffer in D_2_O and manually injected into the flow cells. Polymers were incubated for 2 h, prior to flushing with 15 mL of deuterated buffer at a flow rate of 1 mL min^–1^ to remove excess polymer. The structure of the bilayers following polymer interaction was then characterized in D_2_O, H_2_O, AuMW, and SiMW.

### Neutron Reflectometry Data Analysis

NR data was analyzed using RasCAL 2019 v1.2 (https://github.com/arwelHughes/RasCAL_2019/releases/tag/v1.2). A custom model was defined describing a series of slabs, each with a thickness, roughness, scattering length density (SLD), and roughness, flanked by two bulk phases corresponding to Si and solvent. A total of 9 interfacial slabs were used to analyze the data. The first four slabs after the Si bulk phase correspond to SiO_*x*_, permalloy, Au, and the *N*-(2-hydroxyethyl)-16-mercaptohexadecanamide SAM, and their structural parameters corefined against all data sets collected on an individual substrate before and after polymer interaction. The SLD of the Si substrate and the Au film were fixed (Table S1). The SLD of SiO_2_, permalloy, and the SAM were fitted, accounting for uncertainties in precise compositions and/or molecular volumes, and also corefined across all data sets collected on a given substrate. The pristine floating asymmetric bilayer, prior to SNAP interaction, was analyzed as 5 slabs, corresponding to a solvent layer between the SAM and the bilayer, inner headgroups, inner tails, outer tails, and outer headgroups. The SLD of each component was fixed (Table S2). In the case of the LPS core oligosaccharide, due to the presence of exchangeable protons, the SLD changes as a function of the bulk D_2_O content. This can be calculated based on the known SLD of the core oligosaccharide in D_2_O (SLD_LPS,D_2_O_ = 4.28 × 10^–6^ Å^–2^) and H_2_O (SLD_LPS,H_2_O_ = 2.01 × 10^–6^ Å^–2^) and the SLD of the bulk solvent (SLD_solv_) as

$${SLD}_{LPS}=\left(\frac{{SLD}_{LPS,D_{2}O}-{SLD}_{LPS,H_{2}O}}{{SLD}_{D_{2}O}}\times \left({SLD}_{solv}-{SLD}_{H_{2}O}\right)\right)+{SLD}_{LPS,H_{2}O}$$

The net SLD of each layer was calculated according to the fitted asymmetry, defined as the ratio of DPPC to LPS in the inner leaflet, χ_PC_

$${SLD}_{Inner\;Headgroups}=\chi_{PC}{SLD}_{PC\;Headgroup}+\left(1-\chi_{PC}\right){SLD}_{Core\;Oligosaccharide}$$

$${SLD}_{Outer\;Headgroups}=\chi_{PC}{SLD}_{Core\;Oligosaccharide}+\left(1-\chi_{PC}\right){SLD}_{PC\;Headgroup}$$

The SLDs of the inner and outer tails were calculated in the same way, substituting SLD_PCHeadgroup_ and SLD_Core Oligosaccharide_ for SLD_PCTails_ and SLD_LPSTails_, respectively. Due to both leaflets of the bilayer being physically coupled, a single roughness value was fitted to all slabs. The hydration of both tail leaflets was coupled, and headgroup hydration parameters were independently fit. Thickness values were fitted to each slab. These fitted parameters were corefined across the four isotopic contrasts collected for the pristine asymmetric bilayer. Data corresponding to the floating bilayers after SNAP interaction were analyzed in the same way, except for the asymmetry parameter. In this case, it was assumed that SNAP interaction would not cause an increase in asymmetry, so the volume fraction of DPPC in the inner leaflet was permitted to decrease only compared to the equivalent parameter prior to SNAP interaction. The bulk solvent SLD was independently fit for each contrast, accounting for solvent exchange inefficiency and H/D exchange.

Initial fits were obtained using a Simplex least-squares minimization algorithm incorporated in RasCAL. The error associated with each fitted parameter was estimated using a Bayesian Markov Chain Monte Carlo (MCMC) implemented within RasCAL. In all cases, a uniform prior distribution was assumed. 10^5^ burn-in iterations were performed for location of the global minimum, which were then discarded before performing 5 × 10^5^ iterations used for determination of the posterior distribution for each parameter and subsequent calculation of parameter confidence intervals. In order to reduce the computational resource required, 10^3^ random samples were taken from the chain for calculation of confidence intervals associated with fits to the reflectivity data, SLD profiles, and volume fraction profiles.

## Results and Discussion

This study focuses on two block copolymer SNAPs, both composed of a 7:3 ratio of hydrophobic pNIPAM to cationic pAEAM. These SNAPs vary in molecular weight and block segmentation: a diblock copolymer, p­(NIPAM_35_-*b*-AEAM_15_) (a-D50), and a triblock copolymer, p­(NIPAM_35_-*b*-AEAM_30_-*b*-NIPAM_35_) (a-T100-1). The synthesis, characterization, and promising antimicrobial performance and biocompatibility of these SNAPs have been previously reported, where the minimum inhibitory concentrations (MIC) of a-D50 and a-T100-1 against *P. aeruginosa* LESB578 are 4.7 and 9.7 μM, respectively, in cationic adjusted Muller Hinton media (caMHB), as shown in Figure 1.^40^ Interestingly, while the antimicrobial potency is maintained in synthetic cystic fibrosis sputum media, it is lost in synthetic wound fluid; an important observation regarding the future clinical application of these materials, despite the underlying causes being poorly understood. Here, we determine the mechanism of action of these two polymers, allowing the identification of specific modes of interaction with the bacterial envelope, which is important for both the application of these materials and the future design of SNAPs with well-defined and predictable mechanisms of action.

### Effect of SNAP Exposure on the Cell Morphology of Gram-Negative *P. aeruginosa* LESB58

In order to uncover structure–activity relationships, we first investigated the effect of SNAP exposure on the cell morphology and envelope integrity of *P. aeruginosa* LESB58. Scanning Electron Microscopy (SEM) was used to image the cell surface morphology of the individual cells. Micrographs of untreated cells show the characteristic “rough” appearance of the cell surface associated with the rough LPS phenotype (Figure 2a). Upon treatment with either a-D50 or a-T100-1 at 2 × MIC, a reduction in the viable cell counts was observed relative to the unexposed control (Figure S1a). Correspondingly, SEM imaging shows clear effects on the cell surfaces of the treated cells. In the case of a-D50 treatment, damaged and agglomerated cells could be observed, and a subset of cells appeared lysed with visible cytoplasmic leakages. In the case of a-T100-1 treatment, an increase in the roughness and noticeable wrinkles in the surface of the cell could be seen. In contrast to a-D50, no cell lysis could be observed among the imaged cells (Figures 2a and S1b). These data suggest that while both a-D50 and a-T100-1 disrupt the Gram-negative cell envelope, they do so to differing extents, where a-D50 induces more pronounced membrane damage, consistent with its lower MIC compared to a-T100-1.

**Figure 2:**
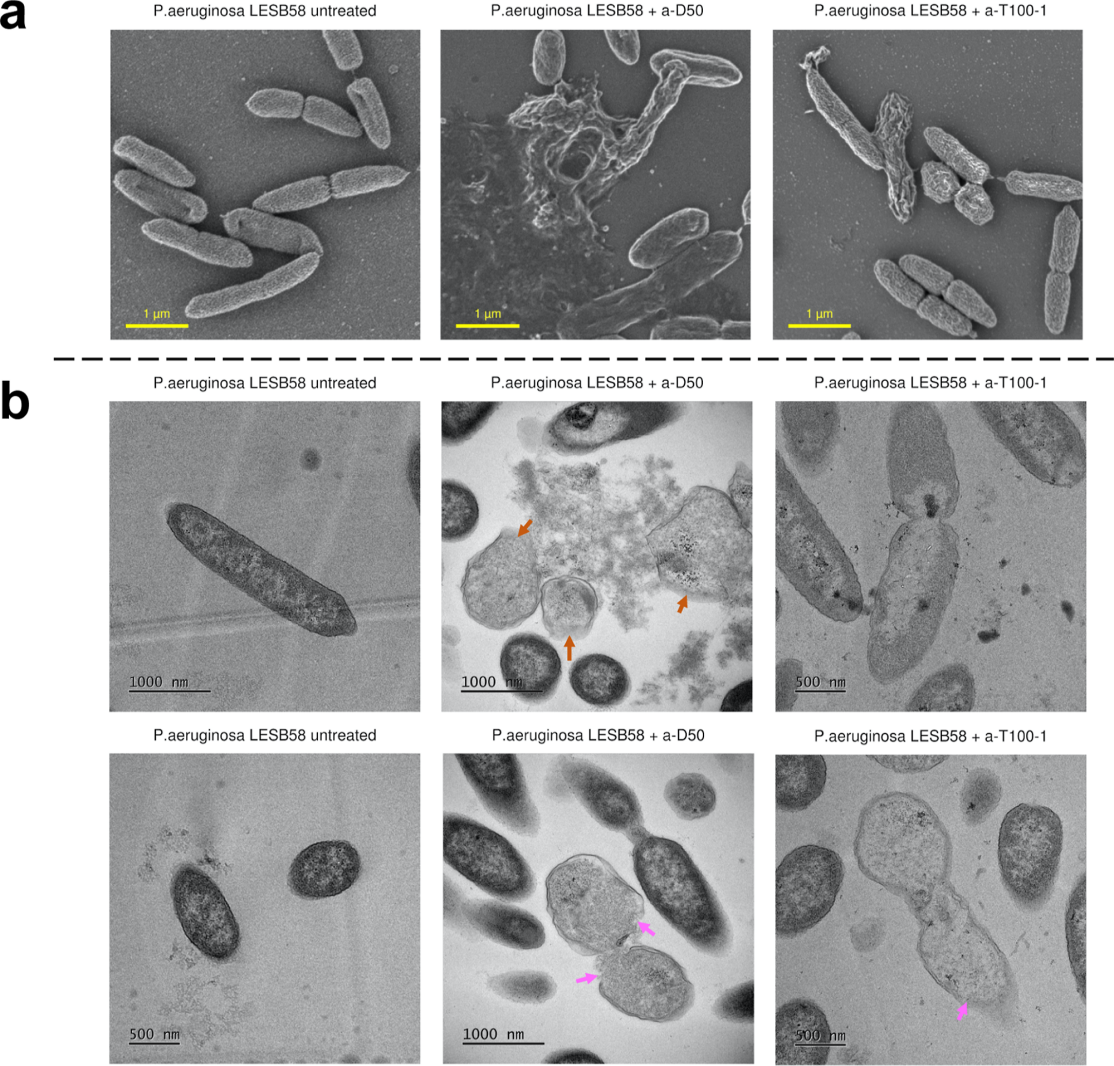
Scanning and transmission electron microscopy images show morphological defects associated with the cell membranes of *P. aeruginosa* following SNAP treatment. (a) Representative scanning electron micrographs of *P. aeruginosa* LESB58 (untreated), *P. aeruginosa* LESB58 treated with a-D50 at 2 × MIC concentration, and *P. aeruginosa* LESB58 treated with a-T100-1 at 2 × MIC concentration (from left to right). (b) Representative transmission electron micrographs of *P. aeruginosa* LESB58 (untreated), *P. aeruginosa* LESB58 treated with a-D50 at 2 × MIC concentration, and *P. aeruginosa* LESB58 treated with a-T100-1 at 2 × MIC concentration (from left to right).

To further investigate the effects of polymer exposure at the subcellular level, we employed transmission electron microscopy (TEM). The high resolution of TEM, combined with ultrathin sectioning, enabled clear visualization of the inner and outer membranes, as well as intracellular structures. Treated cells were imaged following exposure to each SNAP at 2 × MIC (Figure 2b), allowing us to assess architecture-specific damage across the bacterial envelope. Similarly to the SEM images discussed above, clear damage to cell envelopes could be identified in subpopulations of SNAP-exposed cells in comparison with nonexposed control cells (Figures 2b and S2). In cells exposed to a-D50, extensive damage was evident. Lysed cells could be clearly identified with cytoplasmic contents leaking from large ruptures spanning both inner and outer membranes. This was accompanied by reduced electron density of the cell interior, indicative of cytoplasmic loss (Figure 2b, see orange arrows). Together with SEM data, these results strongly suggest that a-D50 exposure leads to the lysis of a subset of *P. aeruginosa* LESB58 cells, through either direct membrane interaction or disruption of osmotic pressures across the cell envelope or a combination of both. In contrast, cells treated with a-T100-1 displayed more moderate morphological changes. While membrane perturbation and cytoplasmic leakage were observed (Figure 2b, purple arrows; Figure S2), no fully ruptured cells were observed. The reduced electron density within the cytoplasm suggests increased permeability of the envelope but not through complete cell rupture. These findings support the conclusion that while both SNAPs disrupt the Gram-negative cell envelope, a-D50 exerts a more severe, lytic effect compared to the subtler, nonlytic disruption caused by a-T100-1.

Further characterization by atomic force microscopy (AFM) was undertaken by providing high-resolution topography images in liquid on *P. aeruginosa* LESB58 cells fixed with glutaraldehyde. Topography images of untreated cells revealed highly textured surfaces, with numerous rough protrusions measuring approximately 26–46 nm in height and 30–50 nm in width (Figure 3a,d, green arrow). These features likely correspond to aggregates of rough LPS layered atop a smoother background, likely corresponding to the OM. This observation corroborates the results from the SEM images and confirms that the highly rough features are not drying artifacts caused by the SEM sample preparations but does not exclude the possibility of glutaraldehyde-induced LPS aggregation, as this is used in both AFM and SEM experiments. *P. aeruginosa* LESB58 treated with a-D50 prior to fixing seems to have lost its rough LPS layer. A logical assumption is that the smoother surface could correspond to the outer lipid bilayer without LPS features (Figure 3b,e). Considering the height distributions (expressed as standard deviation) from the whole bacteria (Figure 3i), we observe that the cells of *P. aeruginosa* LESB58 treated with a-D50 have a significantly lower cell height value (633 ± 33 nm) compared with untreated cells (737 ± 87 nm; *p*_Control‑aD50_ (*) = 0.0193) and a-T100-1 treatment (667 ± 32 nm; *p*_aD50‑aT1001_ (*) = 0.0489). We hypothesize that this effect can be ascribed to the cell envelope being thinner, which suggests that *P. aeruginosa* LESB58 cells treated with a-D50 have likely lost the rough LPS layer, the outer lipid bilayer, and probably the peptidoglycan layer, leaving the inner lipid bilayer exposed, which appears smoother than the original OM (Figure 3b,e). As shown by electron microscopy, a-D50 treatment induces cell rupture and loss of cytoplasmic material, which, in conjunction with sample fixation procedures, may additionally contribute to the observed thinning. Finally, the *P. aeruginosa* LESB58 cells treated with a-T100-1 prior to fixing seem to have lost the LPS features, and the outer lipid bilayer is exposed with visible perforations and scars of 15–50 nm in diameter and 20–30 nm in thickness (Figure 3c,f, see purple dotted arrow).^44^ Compared to AFM studies of the surface of Gram-positive bacteria with an exposed peptidoglycan cell wall, the characteristic mesh-like architecture of peptidoglycan is not visible here,^45^ thus suggesting that in contrast to a-D50, the a-T100-1 polymer primarily damages the lipid bilayer membranes through pore formation through the OM. As shown in Figure S7i,j, we measured the average depth profiles of the LPS patches (around 33 nm) above a uniform layer (lipidic bilayer) where pores around 25 nm were observed in the treatment group (a-T100-1) in comparison with the untreated control.

**Figure 3:**
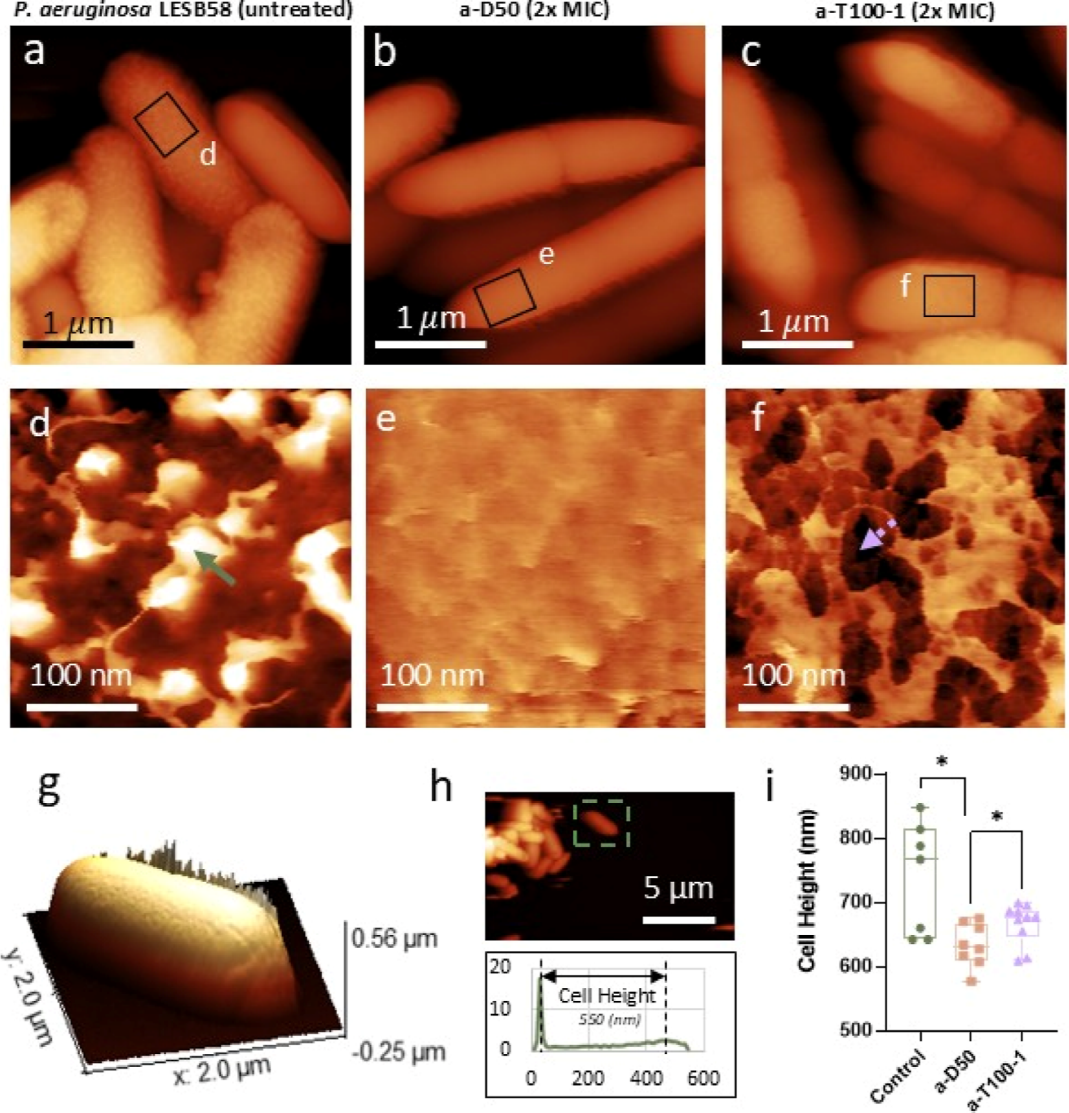
High-resolution AFM height topography images provide evidence of SNAPs targeting the OM of *P. aeruginosa*, compromising OM integrity. (a) *P. aeruginosa* LESB58 untreated cells. (b) *P. aeruginosa* LESB58 cells treated with a-D50 at 2 × MIC concentration. (c) *P. aeruginosa* LESB58 cells treated with a-T100-1 at 2 × MIC concentration. *Z* scale of a–c = 1.3 μm., (d–f) High-resolution images zoomed from (a–c), respectively, showing details of the nanometric architecture of the cell envelop of each sample (green solid arrow points to LPS feature; dotted purple arrow points to OM perforation). *Z* scale of images d–f = 40 nm. (g) Three-dimensional representation of a *P. aeruginosa* LESB58 untreated cell in liquid with AFM. (h) Height image showing an overview of the cells on the surface in liquid, inset: height distribution from a single cell area including the background (dashed green square). (i) Box plot showing the height of different cells in liquid for each treatment and control.

Moreover, the height distributions from the whole bacteria (Figure 3h,i) show that *P. aeruginosa* LESB58 treated with a-T100-1 have a thicker cell envelope than *P. aeruginosa* LESB58 treated with a-D50, while still reduced compared to untreated cells. This is consistent with the OM not being completely removed but instead a-T100-1 forming pores through the OM, abolishing osmotic control across the cell envelope and leading to the loss of intracellular material, also corroborated by EM data.

Collectively, SEM, TEM, and AFM data reveal distinct but potent modes of action for the two SNAPs investigated here. a-D50 exposure results in catastrophic membrane disruption and cell lysis, indicative of a highly aggressive bactericidal mechanism. In contrast, the a-T100-1 treatment compromises the structural integrity and permeability of the cell envelope without causing whole cell lysis, suggestive of the formation of nanoscale pores. While this may appear less severe from a morphological perspective, the increased permeability of the cell envelope is nonetheless lethal, as osmotic and potentiometric gradients are impaired and intracellular material is lost, all resulting in cell death.

### Ammonium-Containing SNAPs Bind to LPS

The morphological effects observed with SEM, TEM, and AFM point toward a strong interaction of both a-D50 and a-T100-1 with the outer membrane of *P. aeruginosa* LESB58. Previously, we showed a lack of toxicity toward mammalian cells for both polymers,^40^ suggesting that antimicrobial activity does not arise through nonspecific lipophilic interactions. Instead, as the antimicrobial activity of these ammonium-containing polymers seems to arise through an interaction with the OM, we hypothesize that there may be a specific interaction with LPS as the major constituent of the outer leaflet of the outer membrane of Gram-negative bacteria. To investigate this hypothesis, we assessed whether a-D50 and a-T100-1 directly bind to the LPS of *P. aeruginosa* LESB58 using a fluorescence-based displacement assay with BODIPY-TR-cadaverine, a cationic fluorescent probe known to bind LPS.^46^ The rough LPS of *P. aeruginosa* LESB58 was purified by following the protocol described by Darveau et al.^47^ Polymer binding was quantified by measuring the increase in fluorescence intensity, corresponding to the displacement of BODIPY-TR-cadaverine upon polymer binding.

As observed in Figure 4, a-D50 showed significant dye displacement due to binding to LPS even below the MIC value, and greater LPS binding could be observed in a dose-dependent manner (Figure 4a). In contrast, a-T100-1 exhibited lower dye displacement, with statistically significant displacement observed only at MIC and 2 × MIC concentrations (Figure 4b). The lower dye displacement observed by a-T100-1 in comparison with a-D50 does not exclude the possibility that the compound still binds to LPS. Polymer interaction with a different binding site to the dye could explain the lower dye displacement while still binding to LPS. Nevertheless, the two compounds exhibited a distinct behavior against LPS, and the lower affinity of a-T100-1 for LPS is consistent with the less severe morphological effects on cell membranes observed by EM and AFM. These results support the hypothesis that the antimicrobial activity of both SNAPs involve specific interactions with LPS but also suggest that the strength and extent of this interaction are influenced by block architecture—potentially correlating with the more pronounced morphological damage and lower MIC observed for a-D50.

**Figure 4:**
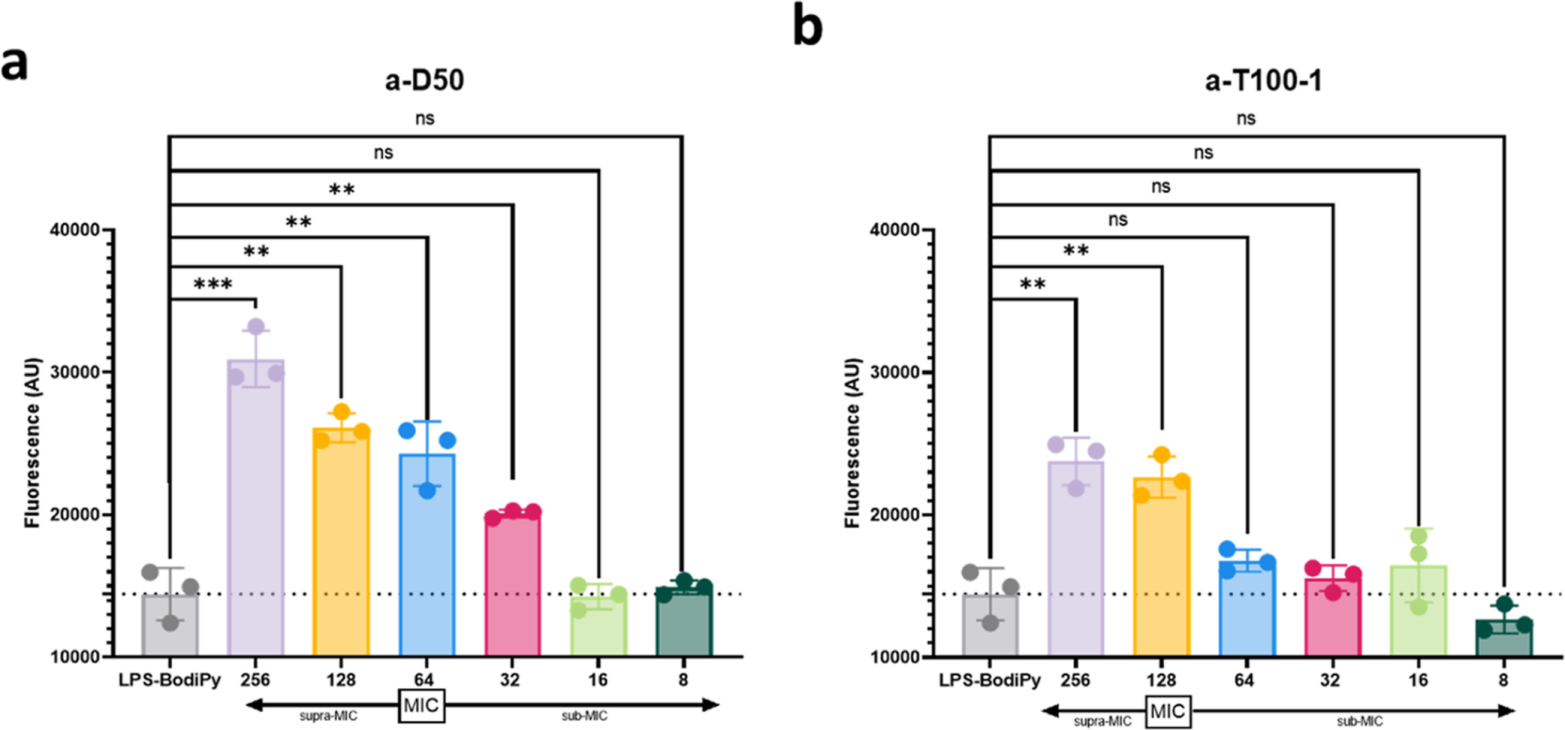
SNAPs exhibit binding affinity for LPS extracted from *P. aeruginosa*. The binding affinity of (a) a-D50 and (b) a-T100-1 to the LPS from *P. aeruginosa* LESB58 was determined by the BODIPY-TR-cadaverine displacement method, as described in Supporting Information. Fluorescence intensity was monitored at an excitation wavelength of 580 nm and an emission wavelength of 620 nm. The graphs were derived from mean values of three independent replicates. *t* tests were performed to compare the fluorescence intensity of the untreated controls with the respective drug treatment.

### Neutron Reflectometry Determines the Polymer-Induced Structural Disruption to the Gram-Negative Outer Membrane

Given the clear and specific disruption of the *P. aeruginosa* LESB58 OM by a-D50 and a-T100-1 observed via microscopy and their confirmed binding to LPS in vitro, we next aimed to probe these interactions at the molecular level. Neutron reflectometry (NR) is a powerful technique for resolving the structure of model lipid bilayers with subnanometer precision.^48–50^ Briefly, NR is based on the specular reflection of a collimated neutron beam from flat interfaces and measures the intensity of the reflected beam as a function of momentum transfer (*Q*_*z*_ = 4π sin θ/λ, where 2θ is the angle of reflection and λ is the neutron wavelength). Neutrons reflect and refract through layers of different refractive indices at the interface, interfering with one another, leading to a modulation of the intensity (analogous to observing a rainbow effect from thin-film interference in the reflection of visible light from an oil film on water). The position and intensity of the characteristic Kiessig interference fringes provides information on the interfacial structure perpendicular to the interface, allowing the determination of depth-dependent density profiles. A particular strength of NR lies in the isotopic sensitivity of neutrons to hydrogen isotopes, protium and deuterium. We exploit this by using differential labeling of different components across the interface as well as the bulk solvent to provide contrast sufficient to resolve the complex compositional distributions with a high level of confidence. For a more detailed description of NR, we direct the reader to the following refs 48 and 50 . Over recent years, there have been robust model membrane systems developed, which accurately mimic the physicochemical properties of the Gram-negative outer membrane, which are amenable for structural characterization with reflectometry.^51–53^ These platforms have proven instrumental in elucidating the mechanisms of action of various antimicrobial compounds,^18,21,39,49,54–56^ and here, we employ them to investigate how block architecture influences SNAP interaction with the OM at the molecular level.

Building on previous work,^52^ we prepared floating asymmetric lipid bilayers that mimic the OM of *P. aeruginosa* LESB58. These were deposited on gold-coated substrates functionalized with a hydrophilic *N*-(2-hydroxyethyl)-16-mercaptohexadecanamide self-assembled monolayer (SAM) to provide a hydrophilic support. To utilize the isotopic contrast capabilities of neutron reflectometry, we constructed bilayers with an inner leaflet of tail-deuterated dipalmitoyl-^2^H_62_-*sn*-glycero-3-phosphocholine (dDPPC) deposited via Langmuir–Blodgett (LB) transfer, and an outer leaflet composed of hydrogenous rough LPS deposited by Langmuir–Schaeffer (LS) transfer, closely replicating the asymmetry and physicochemical properties of the *P. aeruginosa* LESB58 OM.

The structure of these pristine bilayers, before SNAP exposure, were characterized under physiological buffer conditions (20 mM HEPES, 2 mM CaCl_2_, pH 7.4) using NR in four isotopic contrasts: D_2_O, gold-matched water (AuMW; 75% D_2_O/25% H_2_O), silicon-matched water (SiMW; 38% D_2_O/62% H_2_O), and H_2_O (Figure 5a and 6a). Simultaneous fitting of the NR data across these contrasts yielded scattering length density (SLD) profiles (Figure 5b,c) consistent with the successful formation of highly asymmetric bilayers (≥79%) with high coverage (≥85%). These results demonstrate the robustness and reproducibility of the bilayer deposition process, with structural parameters in agreement with previous studies (Table 1, full parameters in Tables S1 and S2).^52^

**Figure 5:**
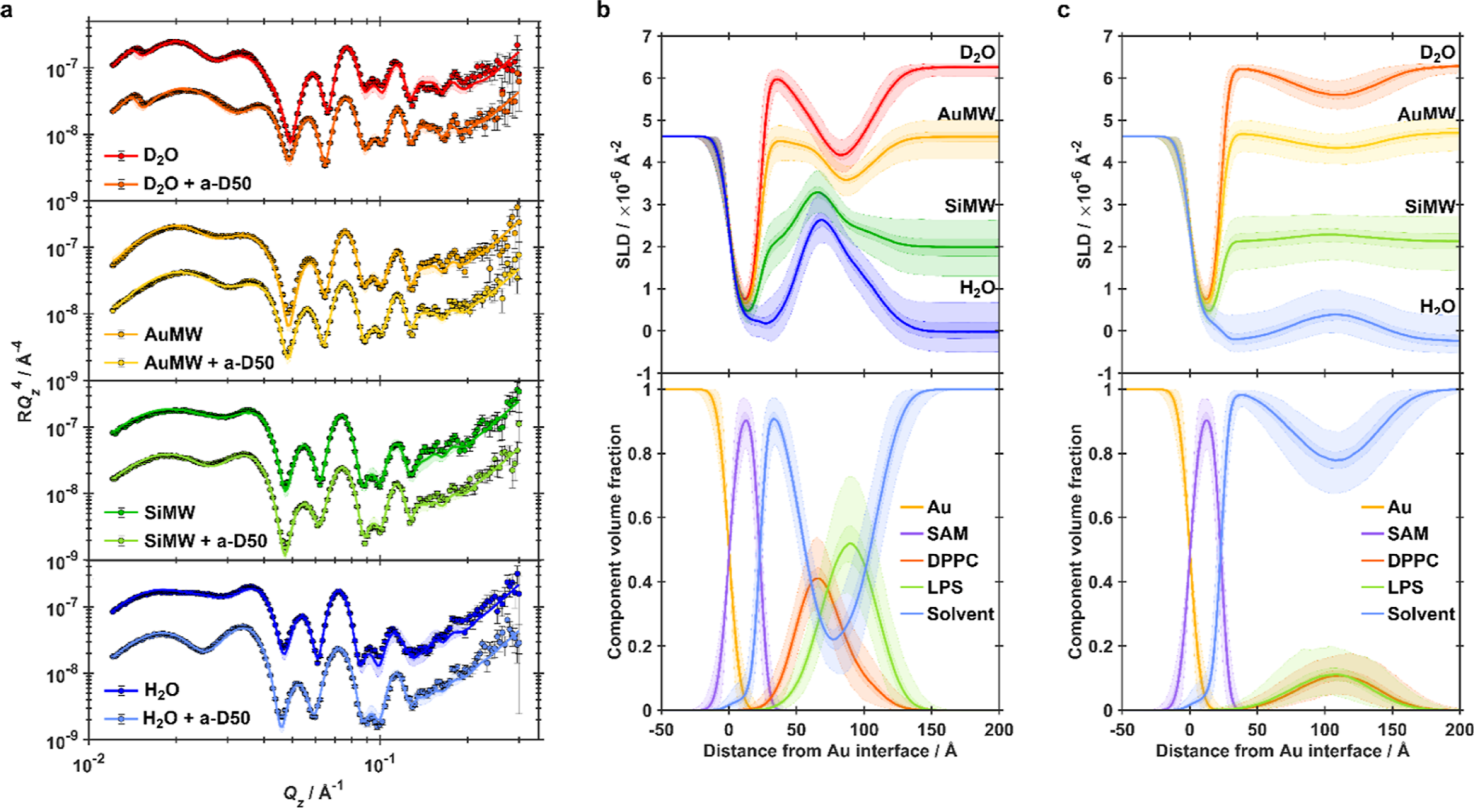
Neutron reflectometry shows exposure to 0.5 μM a-D50 acts to severely reduce the surface coverage of planar floating membrane models of the *P. aeruginosa* OM, consistent with OM solubilization. (a) NR data (points) and fits (lines) for a floating asymmetric dDPPC/hLPS bilayers deposited on a SAM-functionalized gold surface, before and after interaction with a-D50. The bilayer was characterized in D_2_O (red data), gold-matched water (gold data), protein-matched water (green data), and H_2_O (blue data) solution isotopic contrasts. (b) SLD profiles (top panel) and component volume fraction profiles (bottom panel) describing the structure of the pristine floating asymmetric dDPPC/hLPS bilayers prior to interaction with a-D50. (c) SLD profiles (top panel) and component volume fraction profiles (bottom panel) describing the structure of the dDPPC/hLPS bilayers after interaction with a-D50. Throughout, shaded regions represent the 68% (1σ) and 95% (2σ) confidence intervals estimated from Bayesian analysis.

**Figure 6:**
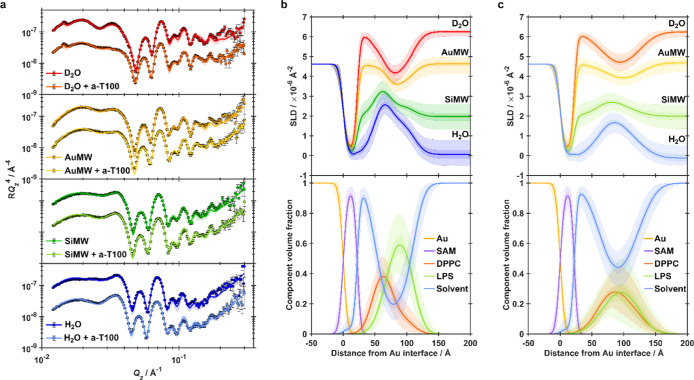
Neutron reflectometry shows exposure to 0.5 μM a-T100-1 leads to a reduction in surface coverage and a loss of asymmetry of planar floating membrane models of the *P. aeruginosa* OM, consistent with pore formation. (a) NR data (points) and fits (lines) for floating asymmetric dDPPC/hLPS bilayers deposited on a SAM-functionalized gold surface, before and after interaction with a-T100-1. The bilayer was characterized in D_2_O (red data), gold-matched water (gold data), protein-matched water (green data), and H_2_O (blue data) solution isotopic contrasts. (b) SLD profiles (top panel) and component volume fraction profiles (bottom panel) describing the structure of the pristine floating asymmetric dDPPC/hLPS bilayers prior to interaction with a-T100-1. (c) SLD profiles (top panel) and component volume fraction profiles (bottom panel) describing the structure of the dDPPC/hLPS bilayers after interaction with a-D50. Throughout, shaded regions represent the 68% (1σ) and 95% (2σ) confidence intervals estimated from Bayesian analysis.

**Table 1:** Key Structural Parameters Obtained by Fitting NR Data of Floating Asymmetric dDPPC/hLPS Bilayers Deposited on the TAAA-Functionalized Substrates, before and after Interactions with a-D50 and a-T100-1^b^

	a-D50		a-T100-1	
parameter	pristine bilayer	bilayer following polymer interaction	pristine bilayer	bilayer following polymer interaction
water gap thickness/Å	27_–2_^+2^	42_–4_^+3^	25_–3_^+2^	35_–6_^+4^
inner headgroup thickness/Å	8_–2_^+3^	36_–6_^+6^	8_–2_^+3^	16_–6_^+8^
inner tails thickness/Å	16_–1_^+1^	9_–3_^+3^	15_–1_^+1^	12_–6_^+3^
outer tails thickness/Å	8_–2_^+2^	9_‑3_^+3^	8_–2_^+2^	11_–3_^+3^
outer headgroup thickness/Å	32_–4_^+3^	27_–10_^+9^	34_–4_^+3^	37_–6_^+5^
asymmetry/%^a^	68_–10_^+9^	–16_–27_^+26^	71_–10_^+9^	20_–67_^+26^
bilayer coverage/%	88_–4_^+5^	28_–7_^+9^	87_–6_^+5^	80_–8_^+7^
bilayer roughness/Å	14_–1_^+1^	21_–2_^+2^	14_–1_^+1^	21_–2_^+1^

a Bilayer Asymmetry is here defined as the difference between the volume fractions of dDPPC and hLPS in the inner leaflet, as a percentage of the total lipid volume fraction in that leaflet, i.e., $asymmetry=100\left(\chi_{v,dDPPC}-\chi_{v,hLPS}/\chi_{v,dDPPC}+\chi_{v,hLPS}\right)$ . The absolute volume fractions of each component are provided in Tables S1 and S2.

b Quoted values represent the median of the posterior distribution for each parameter, and the uncertainties represent the 68% (1σ) confidence interval.

To probe the molecular effects of polymer interaction with the OM-mimetic bilayers, both a-D50 (Figure 5) and a-T100-1 (Figure 6) were incubated with the floating membranes at subinhibitory concentrations (0.5 μM, below the MIC for both polymers) for 2 h at 37 °C. This approach was designed to capture intermediate structural effects without triggering complete membrane dissolution. At or above MIC concentrations, polymer exposure consistently resulted in full membrane removal from the SAM-functionalized gold substrate, precluding structural analysis. Following incubation, the membranes were rinsed with D_2_O and characterized using NR across four isotopic contrasts.

For a-D50, notable changes in the reflectivity curves were observed, including a pronounced shift of the Kiessig fringe minima to lower *Q*_*z*_ was observed in the resulting NR data, most pronounced in D_2_O and H_2_O. This shift is indicative of an overall increase in the thickness of the interfacial structure (Figure 5c). Model fitting of these NR data to produce SLD profiles describing the resultant interfacial structure revealed several striking effects: a striking decrease in surface coverage from ∼88% to ∼25% combined with almost complete abolishment of bilayer asymmetry. While some uncertainty in model parameters remains due to the roughened and partially disrupted bilayer (Figure S4), the data clearly show that a-D50 induces large-scale structural disintegration of the membrane.

These data suggest that the diblock architecture of a-D50 imparts surfactant-like behavior, effectively solubilizing lipids from the bilayer. The resulting patchy and irregular membrane leads to increased interfacial roughness. Despite this disruption, the residual membrane thickness (∼78 Å) thickened slightly compared with the pristine membrane (∼63 Å). Although direct modeling of polymer distributions throughout the membrane was inconclusive, we can hypothesize that polymer embedding would lead to a thickening of the LPS core oligosaccharide, which is now present in both leaflets, supported by the fitted value for the inner headgroup thickness (36 Å), which is much larger than that expected for solely phosphatidylcholine (Figure 7). Furthermore, recent studies have shown that subtle changes in ionic strength and electrostatic interactions can drastically influence the thickness of the water interlayer in floating planar membranes.^57–59^ Here, the observed loss of asymmetry relocates negatively charged phosphate groups from the LPS closer to the substrate. This, combined with potential cationic polymer binding, could modulate the electrostatic and van der Waals interactions responsible for maintaining the membrane-surface distance, leading to the observed increase in the distance between the SAM and the membrane. This effect, combined with the observed thickening of the membrane, explains the shift in Kiessig fringe minima to lower *Q*_*z*_ noted above.

**Figure 7:**
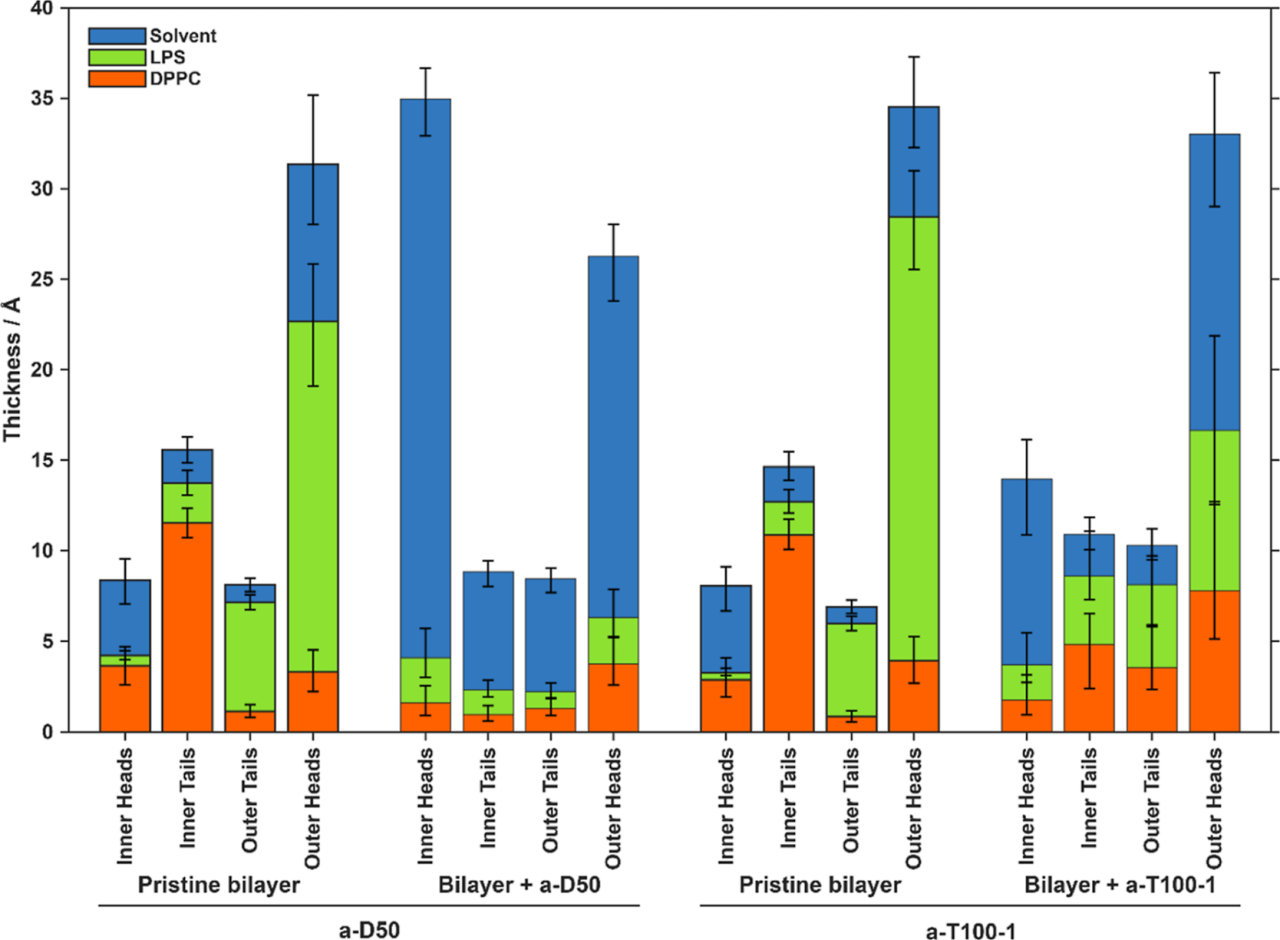
Parameters obtained from fitting neutron reflectometry data indicate that a-D50 leads to a more drastic removal of lipids from Gram-negative bacterial OM models, while both a-D50 and a-T100-1 lead to a loss of asymmetry and a net thickening of the remaining membrane. Total bar heights represent the median layer thicknesses. Each bar is subdivided according to the median volume fraction of each component present within that layer. Error bars represent the 68% (1σ) confidence interval on the volume fraction of each component within the layer.

Following exposure to a-T100-1, NR data revealed more subtle structural alterations to the bilayer than those induced by a-D50 (Figure 6). Simultaneous fitting of the reflectivity data indicated only a slight reduction in bilayer coverage from ∼87% to ∼80%, contrasting starkly with the pronounced membrane loss observed for a-D50. Nevertheless, a-T100-1 induced a comparable loss of bilayer asymmetry and a notable thickening of the inner headgroup region (Table 1 and Figure 7). Combined with an increase in bilayer roughness, these results suggest significant polymer association with the membrane, likely forming pores as observed with AFM, though the complexity of the system prevented definitive modeling of polymer distribution from the NR data alone.

Together, these findings offer crucial insight into the distinct molecular mechanisms of action of these SNAPs. Consistently across all experiments, a-D50 demonstrated a more aggressive and disruptive mode of action than a-T100-1. NR, AFM, and binding data consistently point to a surfactant-like activity of a-D50, likely driven by specific interactions with the LPS core oligosaccharide. This results in solubilization of the outer membrane, destruction of bilayer asymmetry, and a loss of membrane integrity. Apart from OM disruption, AFM did not show any structural features characteristic of peptidoglycan, suggesting that this too may have been disrupted, resulting in a revealed markedly rough residual surface, in agreement with the increased interfacial roughness observed by NR. Whether a-D50 is directly disrupting peptidoglycan remains to be determined. The functional consequences of this envelope disruption are profound. a-D50 exposure leads to a complete collapse of osmotic and electrochemical gradients across the envelope, culminating in catastrophic cell rupture, as observed additionally via TEM and SEM.

In contrast, a-T100-1 exerts its antimicrobial effect through a more subtle mechanism. AFM indicates the formation of discrete pores across the outer membrane. Additionally, NR data confirm the retention of much of the bilayer structure—albeit with abolished asymmetry and ∼10% increased solvent penetration. These structural perturbations compromise the membrane’s permeability barrier and disrupt osmotic and potentiometric homeostasis but do not induce full-scale membrane solubilization or cell lysis. Instead, they result in localized cytoplasmic leakage and membrane ruffling, corroborated by the nanometer changes observed in TEM and SEM images.

These results clearly indicate that the differences in polymer structure between a-D50 and a-T100-1 result in differences in their mechanism of action, and we can speculate as to how these differences in mechanism arise. Our previous study investigated the structure of these polymers in solution and found that at physiological pH, a-T100-1 experiences a stronger aggregation than a-D50. This is likely driven by the longer PNIPAM block, which is hydrophobic at 37 °C. This stronger aggregation, even though observed at a much higher concentration than that investigated here, would result in less solvent-exposed PNIPAM, which may mean a-T100-1 is less able to penetrate into the hydrophobic core of the bilayer, corroborating the results observed here. Counter to this, the shorter PNIPAM block of a-D50 drives less aggregation and is better able to penetrate into the membrane. We hypothesize that a-D50 is able to remove lipid from the membrane by forming stable polymer-lipid hybrid micelles, acting as a macrosurfactant, facilitated by the diblock architecture and the shorter length of the hydrophobic block. As the hydrophobic PNIPAM block leads to less aggregation, individual chains are likely better able to initially adsorb and subsequently interact with membrane components and leave the interface, removing more interfacial material in the process. While a-T100-1 also increases solvent penetration throughout the membrane, we hypothesize that this is due to pore formation rather than lipid removal. It is likely that a reduced ability to penetrate into the bilayer means that a critical concentration of embedded polymer required to induce membrane solubilization is not reached, which is an established thermodynamic mechanism for polymer-driven membrane solubilization.^60,61^

The structural influence of SNAP exposure on the Gram-negative OM appears to be more severe than observed for AMPs. Polymyxin, for example, has been shown to reduce the asymmetry of OM models, similarly to the polymers investigated here, but without the large-scale removal of material and increase in solvent penetration through the membrane.^62^ While there has been evidence reported of pore formation by many AMPs,^63,64^ this is not a universal feature, and they do not appear to induce membrane dissolution.^21,56,65,66^

## Conclusion

This study elucidates at the molecular level the interaction of novel antimicrobial compounds and the bacterial envelope of Gram-negative *P. aeruginosa* (recognized as one of the critical multidrug-resistant ESKAPE pathogens) isolated from a cystic fibrosis patient. We investigated the structural interaction of the polymers with bacterial membranes and found that both SNAPs (a-D50 and a-T100-1) interact with LPS, one of the main components of the OM, with a stronger interaction caused by the small molecular weight compound a-D50. EM revealed damage in the OM and IM of *P. aeruginosa*, showing cell lysis and cytoplasm leakage. Liquid AFM on fixed live cells indicated that both a-D50 and a-T100-1 polymers remove the LPS layer and attack the OM. However, from the topography results and the height distribution, it is inferred that a-D50 also damages the peptidoglycan layer and disrupts the IM, causing the lysis observed occasionally in EM.

By using a synthetic asymmetric lipid bilayer mimicking the OM of Gram-negative bacteria and neutron reflectometry, we investigated the molecular structural interaction of SNAPs with the different components of the synthetic OM. a-D50 showed a more severe effect on the structure of the Gram-negative membrane model in comparison with a-T100-1. a-D50 appears to solubilize lipids from the membrane in a surfactant-like manner, in comparison with a-T100-1, which leads to pore formation and a complete loss of asymmetry, potentially adsorbing to the OM surface.

This report is one of the first examples in the field to reveal the disruption mechanism of SNAPs against Gram-negative bacterial membranes. Even if the polymeric materials have similar chemical moieties and cationic/hydrophobic balances, the distribution of these moieties and overall molecular weight seem to play a crucial role in the mechanism of action of the compounds against bacteria. Importantly, these results indicate that tuning the molecular properties of SNAPS can be used to target different mechanisms of OM disruption of Gram-negative bacteria, including differing extents of lipid removal and polymer adsorption. These findings are crucial for designing the next generation of SNAPs with potent antimicrobial activity, specifically targeting membrane disruption in Gram-negative bacteria. By inducing a “leaky phenotype” in the bacterial cell envelope, the extent of which can be controlled by the block segregation in SNAPS, these compounds could be used to resensitize bacteria to drugs that can no longer penetrate their membrane due to the acquisition of multiple resistance mechanisms. Such a combined therapy with antibiotics that poorly penetrate the Gram-negative cell envelope could be a potent way to aid the ongoing AMR crisis.

## Supplementary Material



## Notes

TEM and AFM images of bacteria after treatment; structural parameters obtained through fitting NR data; and corner plot corresponding to parameters describing the silicon substrate, permalloy, gold, and SAM films of the sample used to investigate the interaction of SNAPS (PDF)
