# Supplementary material for: Assessing the Mechanism of Action of Synthetic Nanoengineered Antimicrobial Polymers against the Bacterial Membrane of Pseudomonas aeruginosa
Source: Biomacromolecules. 2025 Sep 22;26(10):6854–68. doi: 10.1021/acs.biomac.5c01175 (PMC12522136; doi:10.1021/acs.biomac.5c01175)
Supplement: Supplementary file 1 [file bm5c01175_si_001.pdf]

Supporting Information

Assessing the mechanism of action of synthetic nanoengineered antimicrobial polymers against the bacterial membrane of *Pseudemonas aeruginosa*

Ramón Garcia Maset [a], *Laia Pasquina-Lemonche* [b], *Alexia Hapeshi* [c], *Luke A. Clifton* [d], *Jamie K. Hobbs* [b], *Freya Harrison* [e], *Sébastien Perrier* [a,c,g] *, *Stephen C. L. Hall* [d] *

a) Warwick Medical School, University of Warwick, Coventry, CV4 7AL, United Kingdom

b) School of Biosciences, University of Sheffield, Sheffield, S10 2TN, United Kingdom

c) Department of Chemistry, University of Warwick, Coventry, CV4 7AL, United Kingdom

d) ISIS Neutron and Muon Source, Rutherford Appleton Laboratory, Didcot, OX11 0DE, United Kingdom

e) School of Life Sciences, University of Warwick, Coventry, CV4 7AL, United Kingdom

f) School of Mathematical and Physical Sciences, University of Sheffield, Sheffield, S3 7RH, United Kingdom

g) Faculty of Pharmacy and Pharmaceutical Sciences, Monash University, Parkville, Victoria 3052, Australia

* Corresponding authors:
Sébastien Perrier. E-mail: s.perrier@warwick.ac.uk
Stephen C. L. Hall. E-mail: stephen.hall@stfc.ac.uk

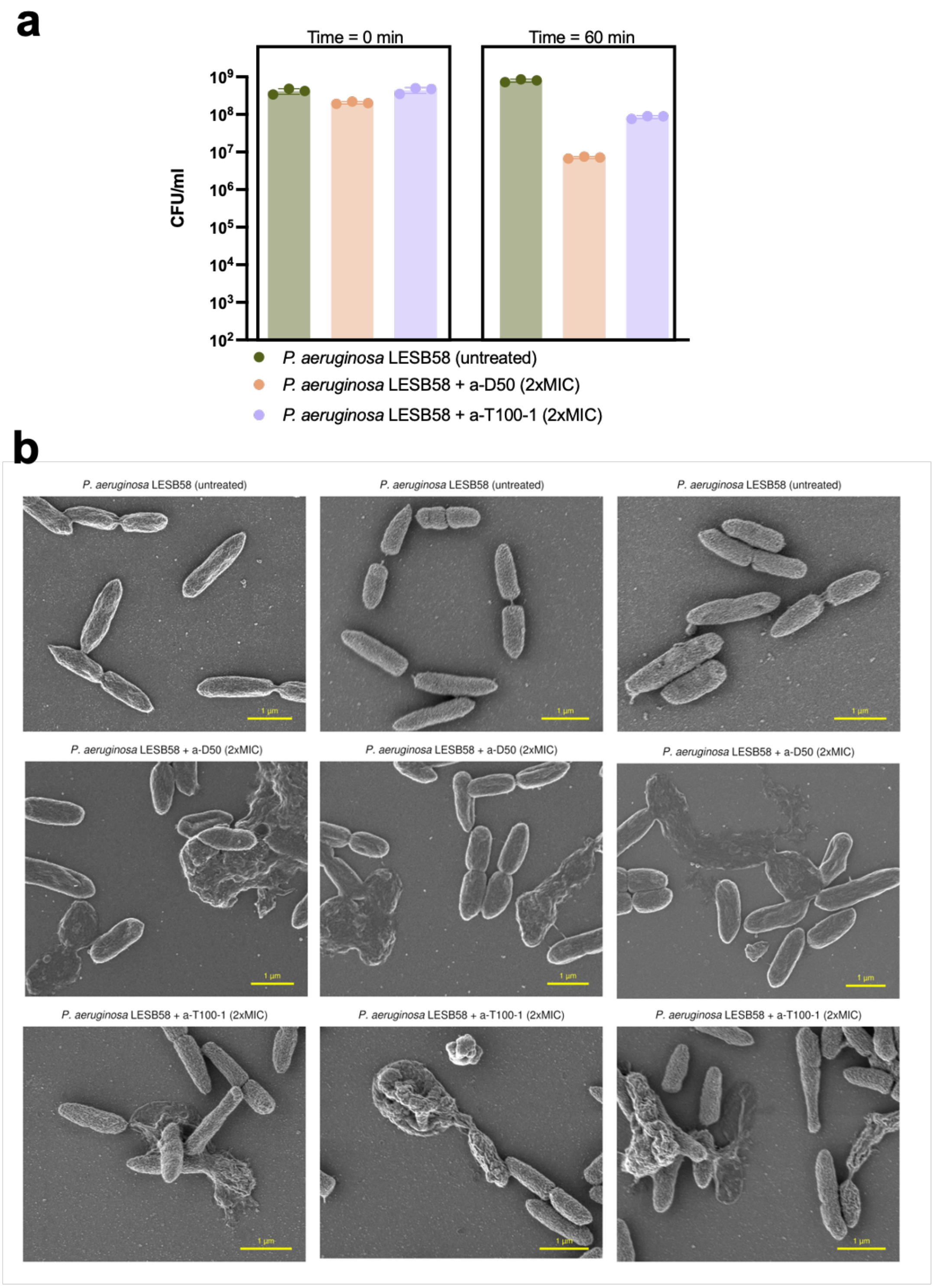

Figure S1. Viable cell counts and representative AFM images of *P. aeruginosa* LESB58 following exposure to SNAPs (**a**) CFU counts of bacteria untreated, exposed to a-D50 at 2×MIC and a-T100-1 at 2×MIC respectively at t=0 and after 60 min treatment. (**b**) Representative scanning electron micrographs of *P. aeruginosa* LESB58 (untreated), *P. aeruginosa* LESB58 treated with a-D50 at 2×MIC concentration and *P. aeruginosa* LESB58 treated with a-T100-1 at 2×MIC concentration.

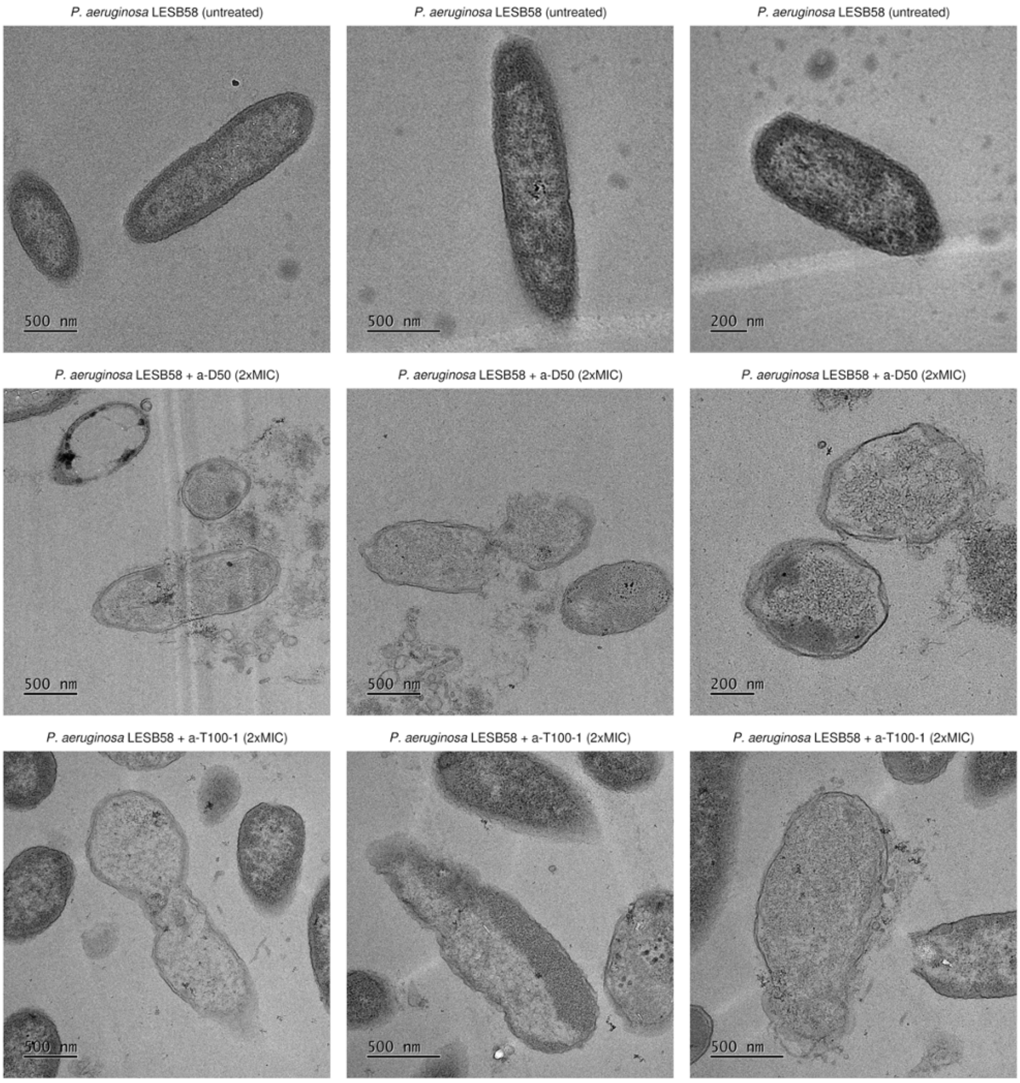

Figure S2. **Representative transmission electron micrographs of *P. aeruginosa* LESB58 following SNAP exposure.** Top row: *P. aeruginosa* LESB58 untreated. Middle row: *P. aeruginosa* LESB58 treated with a-D50 at 2×MIC concentration. Bottom row: *P. aeruginosa* LESB58 treated with a-T100-1 at 2×MIC concentration.

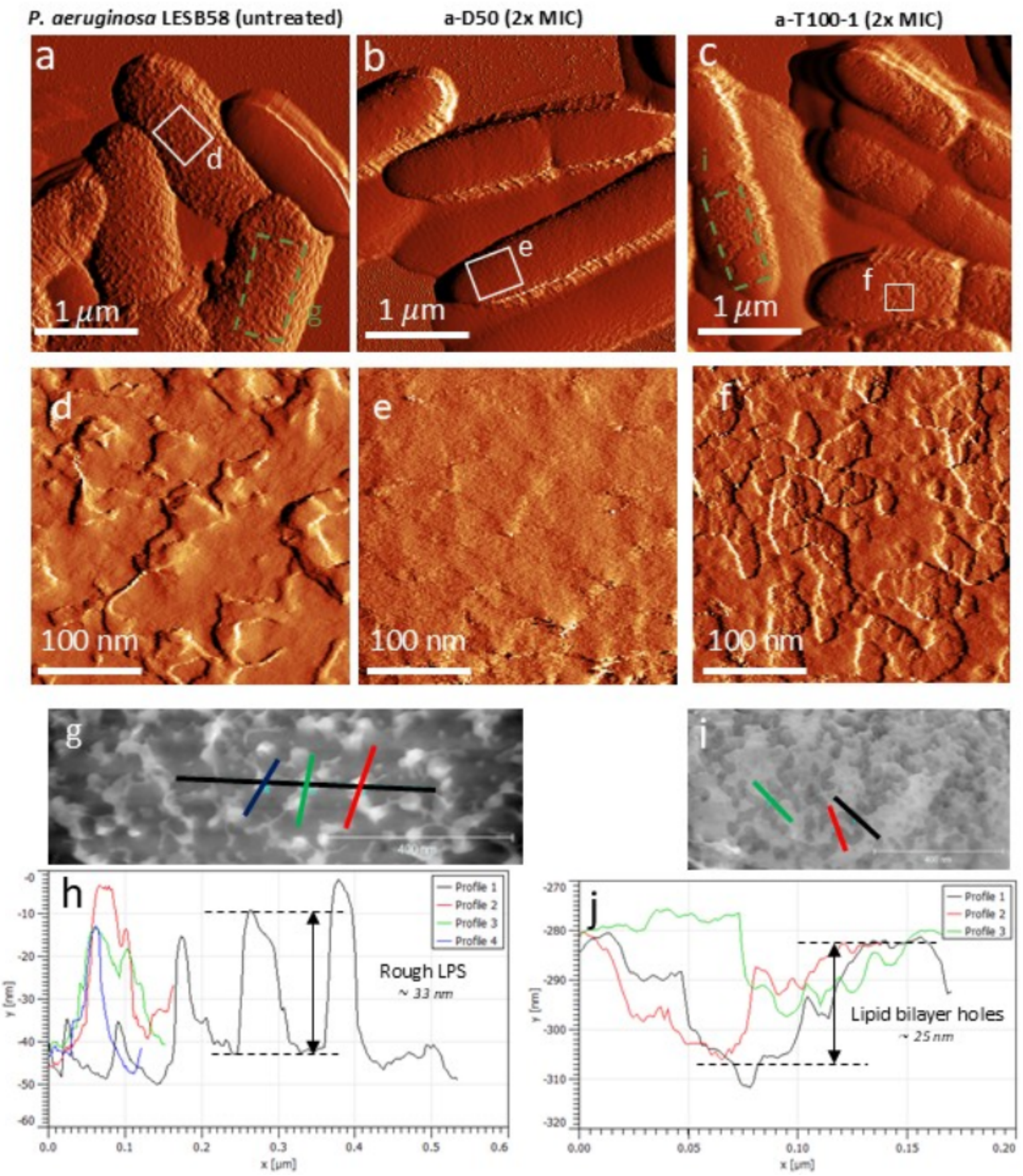

Figure S3. (a-f) the PeakForce error channel taken by the AFM in PeakForce Mode that correspond to Figure 3 (a-f.) This channel highlights the edges of the features in the image and complements the height images presented in Figure 3. (**g**) Heigh image in greyscale from the green dashed box in S3a, highlighting the rough LPS features, four profiles (black, red, green and blue) have been taken on the locations indicated in this image, (**h**) Height profiles from the locations indicated in image S3g, showing an average height of the rough LPS features to be 33 nm. (**i**) Heigh image in greyscale from the green dashed box in S3c, highlighting the porosity in a uniform layer that it is assumed to be a lipid bilayer, three profiles (black, red and green) have been taken on the locations indicated in this image, (**j**) Height profiles from the locations indicated in image S3i, showing an average depth of the pores perforating the flat layer to be 25 nm.

Table S1. Complete list of structural parameters obtained through fitting NR data corresponding to a floating asymmetric bilayer of dDPPC/hRaLPS prepared on a permalloy/gold/SAM-coated silicon substrate before and after incubation with a-D50. Parameters quoted are the median value of the posterior distribution obtained by Bayesian MCMC analysis, and errors represent the 95% (2σ) confidence intervals. Parameter values

within parentheses correspond to the parameter value in the iteration producing the best fit within the MCMC chain. Parameters marked with * were held constant throughout the analysis. Parameters marked with † were not directly fit but derived from fitted parameters and are included here for completeness.

Layer	Thickness / Å	Net SLD / ×10^{-6} Å^{-2}	Component volume fractions / %	Roughness / Å
**SUBSTRATE**				
**Si**	-	2.07*	Si: 100*	$9.5^{+8.1}_{-5.8}$ (4.8)
**SiO_2**	$14.2^{+9.5}_{-12.7}$ (25.4)	$4.10^{+0.80}_{-0.57}$ (3.81)	SiO_2: 100*	$6.4^{+4.2}_{-2.7}$ (3.1)
**Permalloy**	$133.1^{+3.4}_{-3.7}$ (131.6)	$9.03^{+0.08}_{-0.08}$ (8.99)	Permalloy: 100*	$6.1^{+1.5}_{-1.6}$ (5.7)
**Gold**	$174.4^{+2.6}_{-1.9}$ (172.8)	4.62*	Gold: 100*	$5.1^{+3.4}_{-1.9}$ (4.4)
**SAM**	$22.8^{+1.9}_{-2.9}$ (24.8)	$0.26^{+0.39}_{-0.59}$ (0.56)	SAM: 97^{+3}_{-6} (100)†<br>Solvent: 3^{+6}_{-3} (0)	$4.1^{+2.0}_{-1.0}$ (5.6)
**PRISTINE BILAYER**				
**Water Gap**	$28.3^{+6.8}_{-8.7}$ (31.8)	D_2O: $6.18^{+0.22}_{-0.15}$ (6.31)<br>AuMW: $4.57^{+0.30}_{-0.45}$ (4.67)<br>SiMW: $2.04^{+0.66}_{-0.71}$ (2.12)<br>H_2O: $0.11^{+0.65}_{-0.55}$ (0.42)	Solvent: 100*	$13.7^{+3.7}_{-4.9}$ (11.7)
**Inner Headgroups**	$8.8^{+7.9}_{-7.1}$ (5.9)	D_2O: $2.62^{+1.23}_{-0.40}$ (2.55)†<br>AuMW: $2.48^{+0.90}_{-0.29}$ (2.43)†<br>SiMW: $2.30^{+0.34}_{-0.12}$ (2.26)†<br>H_2O: $2.11^{+0.05}_{-0.08}$ (2.14)†	DPPC Headgroups: 36^{+42}_{-32} (74)†<br>LPS Headgroups: 8^{+22}_{-7} (16)†<br>Solvent: 52^{+41}_{-45} (10)	$13.7^{+3.7}_{-4.9}$ (11.7)
**Inner Tails**	$15.2^{+2.6}_{-11.6}$ (16.1)	$5.34^{+1.27}_{-3.91}$ (5.56)†	DPPC Tails: 78^{+17}_{-54} (80)†<br>LPS Tails: 21^{+53}_{-17} (18)†<br>Solvent: $0.4^{+2.9}_{-0.4}$ (2)	$13.7^{+3.7}_{-4.9}$ (11.7)
**Outer Tails**	$9.3^{+6.5}_{-7.1}$ (7.4)	$1.16^{+3.91}_{-1.27}$ (0.93)†	DPPC Tails: 21^{+53}_{-17} (18)†<br>LPS Tails: 78^{+17}_{-54} (80)†<br>Solvent: $0.4^{+2.9}_{-0.4}$ (2)	$13.7^{+3.7}_{-4.9}$ (11.7)
**Outer Headgroup**	$32.9^{+12.2}_{-17.3}$ (35.1)	D_2O: $3.94^{+0.41}_{-1.25}$ (4.06)†<br>AuMW: $3.43^{+0.33}_{-0.89}$ (3.53)†<br>SiMW: $2.65^{+0.26}_{-0.35}$ (2.71)†<br>H_2O: $2.07^{+0.19}_{-0.20}$ (2.17)†	DPPC Headgroups: 13^{+43}_{-11} (10)†<br>LPS Headgroups: 51^{+32}_{-37} (47)†<br>Solvent: 31^{+43}_{-28} (43)	$13.7^{+3.7}_{-4.9}$ (11.7)
**Bulk Solvent**	-	D_2O: $6.18^{+0.22}_{-0.15}$ (6.31)<br>AuMW: $4.57^{+0.30}_{-0.45}$ (4.67)<br>SiMW: $2.04^{+0.66}_{-0.71}$ (2.12)<br>H_2O: $0.11^{+0.65}_{-0.55}$ (0.42)	Solvent: 100*	-
**BILAYER AFTER a-D50 INTERACTION**				
**Water Gap**	$42.4^{+7.0}_{-21.0}$ (38.5)	D_2O: $6.25^{+0.11}_{-0.18}$ (6.27)<br>AuMW: $4.64^{+0.26}_{-0.36}$ (4.67)<br>SiMW: $2.10^{+0.58}_{-0.65}$ (2.01)<br>H_2O: $-0.25^{+0.50}_{-0.28}$ (0.04)	Solvent: 100*	$20.8^{+3.8}_{-10.4}$ (22.9)
**Inner Headgroups**	$33.7^{+17.9}_{-26.4}$ (41.7)	D_2O: $4.05^{+1.13}_{-1.18}$ (4.12)†<br>AuMW: $3.66^{+0.94}_{-0.95}$ (3.73)†<br>SiMW: $3.03^{+0.67}_{-0.57}$ (3.07)†<br>H_2O: $2.48^{+0.38}_{-0.24}$ (2.59)†	DPPC Headgroups: 4^{+13}_{-4} (2)†<br>LPS Headgroups: 7^{+27}_{-7} (3)†<br>Solvent: 86^{+12}_{-30} (94)	$20.8^{+3.8}_{-10.4}$ (22.9)
**Inner Tails**	$8.4^{+8.5}_{-7.5}$ (3.6)	$2.40^{+2.74}_{-2.52}$ (2.31)†	DPPC Headgroups: 9^{+26}_{-8} (22)†<br>LPS Headgroups: 15^{+33}_{-10} (37)†<br>Solvent: 75^{+14}_{-49} (41)	$20.8^{+3.8}_{-10.4}$ (22.9)
**Outer Tails**	$8.9^{+8.2}_{-7.9}$ (3.34)	$4.10^{+2.52}_{-2.73}$ (4.19)†	DPPC Headgroups: 15^{+33}_{-10} (37)†<br>LPS Headgroups: 9^{+26}_{-8} (22)†<br>Solvent: 75^{+14}_{-49} (41)	$20.8^{+3.8}_{-10.4}$ (22.9)
**Outer Headgroup**	$25.9^{+21.6}_{-23.5}$ (10.2)	D_2O: $3.32^{+1.18}_{-1.08}$ (3.30)†<br>AuMW: $3.08^{+0.95}_{-0.86}$ (3.07)†<br>SiMW: $2.69^{+0.62}_{-0.51}$ (2.68)†<br>H_2O: $2.33^{+0.32}_{-0.18}$ (2.40)†	DPPC Headgroups: 15^{+40}_{-13} (14)†<br>LPS Headgroups: 9^{+31}_{-8} (8)†<br>Solvent: 73^{+22}_{-55} (77)	$20.8^{+3.8}_{-10.4}$ (22.9)
**Bulk Solvent**	-	D_2O: $6.25^{+0.11}_{-0.18}$ (6.27)<br>AuMW: $4.64^{+0.26}_{-0.36}$ (4.67)	Solvent: 100*	-

		SiMW: $2.10^{+0.58}_{-0.65}$ (2.01)<br>H_2O: $-0.25^{+0.50}_{-0.28}$ (0.04)		

Table S2. Complete list of structural parameters obtained through fitting NR data corresponding to a floating asymmetric bilayer of dDPPC/hRaLPS prepared on a permalloy/gold/SAM-coated silicon substrate before and after incubation with a-T100-1. Parameters quoted are the median value of the posterior distribution obtained by Bayesian MCMC analysis, and errors represent the 95% (2σ) confidence intervals. Parameter values within parentheses correspond to the parameter value in the iteration producing the best fit within the MCMC chain. Parameters marked with * were held constant throughout the analysis. Parameters marked with † were not directly fit, but derived from fitted parameters and are included here for completeness.

Layer	Thickness / Å	Net SLD / ×10⁻⁶ Å⁻²	Component volume fractions / %	Roughness / Å
**SUBSTRATE**				
**Si**	-	2.07*	Si: 100*	$7.2^{+8.8}_{-3.8}$ (4.6)
**SiO_2**	$11.4^{+10.4}_{-10.6}$ (20.6)	$4.13^{+0.77}_{-0.60}$ (4.81)	SiO_2: 100*	$7.3^{+3.5}_{-3.5}$ (5.6)
**Permalloy**	$134.5^{+3.2}_{-3.8}$ (130.0)	$8.96^{+0.08}_{-0.08}$ (9.00)	Permalloy: 100*	$6.5^{+1.6}_{-1.7}$ (6.3)
**Gold**	$186.1^{+2.1}_{-1.8}$ (186.1)	4.62*	Gold: 100*	$4.5^{+2.5}_{-1.4}$ (3.2)
**SAM**	$21.5^{+2.5}_{-2.5}$ (20.8)	$-0.01^{+0.45}_{-0.43}$ (0.09)	SAM: 97^{+3}_{-7} $(98)^{\dagger}$<br>Solvent: 3^{+7}_{-3} (2)	$3.9^{+1.8}_{-0.8}$ (4.3)
**PRISTINE BILAYER**				
**Water Gap**	$26.3^{+7.7}_{-9.8}$ (32.3)	D_2O: $6.20^{+0.15}_{-0.25}$ (6.12)<br>AuMW: $4.59^{+0.27}_{-0.46}$ (4.75)<br>SiMW: $2.05^{+0.69}_{-0.70}$ (2.36)<br>H_2O: $0.13^{+0.69}_{-0.60}$ (−0.21)	Solvent: 100*	$13.3^{+4.0}_{-5.7}$ (15.3)
**Inner Headgroups**	$7.9^{+8.6}_{-6.8}$ (1.2)	D_2O: $2.54^{+1.54}_{-0.36}$ $(2.44)^{\dagger}$<br>AuMW: $2.43^{+1.13}_{-0.26}$ $(2.37)^{\dagger}$<br>SiMW: $2.25^{+0.45}_{-0.10}$ $(2.24)^{\dagger}$<br>H_2O: $2.12^{+0.06}_{-0.09}$ $(2.10)^{\dagger}$	DPPC Headgroups: 33^{+46}_{-30} $(66)^{\dagger}$<br>LPS Headgroups: 6^{+38}_{-6} $(11)^{\dagger}$<br>Solvent: 55^{+38}_{-48} (23)	$13.3^{+4.0}_{-5.7}$ (15.3)
**Inner Tails**	$14.5^{+3.2}_{-12.0}$ (14.1)	$5.57^{+1.13}_{-4.88}$ $(5.88)^{\dagger}$	DPPC Tails: 70^{+20}_{-59} $(79)^{\dagger}$<br>LPS Tails: 15^{+51}_{-13} $(13)^{\dagger}$<br>Solvent: 12^{+25}_{-11} (8)	$13.3^{+4.0}_{-5.7}$ (15.3)
**Outer Tails**	$8.5^{+8.0}_{-6.9}$ (5.4)	$0.92^{+4.88}_{-1.13}$ $(0.62)^{\dagger}$	DPPC Tails: 15^{+51}_{-13} $(13)^{\dagger}$<br>LPS Tails: 70^{+20}_{-59} $(79)^{\dagger}$<br>Solvent: 12^{+25}_{-11} (8)	$13.3^{+4.0}_{-5.7}$ (15.3)
**Outer Headgroup**	$34.1^{+11.6}_{-15.0}$ (32.5)	D_2O: $4.02^{+0.36}_{-1.22}$ $(4.09)^{\dagger}$<br>AuMW: $3.49^{+0.29}_{-1.12}$ $(3.63)^{\dagger}$<br>SiMW: $2.67^{+0.27}_{-0.44}$ $(2.82)^{\dagger}$<br>H_2O: $2.09^{+0.20}_{-0.22}$ $(1.96)^{\dagger}$	DPPC Headgroups: 14^{+57}_{-12} $(13)^{\dagger}$<br>LPS Headgroups: 67^{+23}_{-55} $(83)^{\dagger}$<br>Solvent: 16^{+37}_{-15} (4)	$13.3^{+4.0}_{-5.7}$ (15.3)
**Bulk Solvent**	-	D_2O: $6.20^{+0.15}_{-0.25}$ (6.12)<br>AuMW: $4.59^{+0.27}_{-0.46}$ (4.75)<br>SiMW: $2.05^{+0.69}_{-0.70}$ (2.36)<br>H_2O: $0.13^{+0.69}_{-0.60}$ (−0.21)	Solvent: 100*	-
**BILAYER AFTER a-T100-1 INTERACTION**				
**Water Gap**	$35.8^{+11.6}_{-28.1}$ (26.3)	D_2O: $6.19^{+0.15}_{-0.27}$ (6.31)<br>AuMW: $4.61^{+0.27}_{-0.53}$ (4.53)<br>SiMW: $1.95^{+0.64}_{-0.65}$ (1.97)<br>H_2O: $-0.11^{+0.69}_{-0.40}$ (−0.01)	Solvent: 100*	$20.7^{+3.9}_{-7.8}$ (17.0)
**Inner Headgroups**	$16.8^{+28.4}_{-15.0}$ (17.8)	D_2O: $3.53^{+1.66}_{-1.00}$ $(2.60)^{\dagger}$<br>AuMW: $3.25^{+1.36}_{-0.80}$ $(2.50)^{\dagger}$<br>SiMW: $2.78^{+0.88}_{-0.47}$ $(2.35)^{\dagger}$<br>H_2O: $2.41^{+0.49}_{-0.22}$ $(2.24)^{\dagger}$	DPPC Headgroups: 11^{+46}_{-10} $(4)^{\dagger}$<br>LPS Headgroups: 15^{+65}_{-14} $(1)^{\dagger}$<br>Solvent: 63^{+34}_{-53} (95)	$20.7^{+3.9}_{-7.8}$ (17.0)
**Inner Tails**	$11.3^{+6.3}_{-10.5}$ (13.6)	$3.60^{+2.33}_{-3.79}$ $(5.84)^{\dagger}$	DPPC Headgroups: 38^{+36}_{-37} $(74)^{\dagger}$<br>LPS Headgroups: 33^{+48}_{-23} $(13)^{\dagger}$<br>Solvent: 23^{+35}_{-20} (13)	$20.7^{+3.9}_{-7.8}$ (17.0)
**Outer Tails**	$11.2^{+6.1}_{-9.1}$ (6.1)	$2.90^{+3.79}_{-2.33}$ $(0.66)^{\dagger}$	DPPC Headgroups: 33^{+48}_{-23} $(13)^{\dagger}$<br>LPS Headgroups: 38^{+36}_{-37} $(74)^{\dagger}$<br>Solvent: 23^{+35}_{-20} (13)	$20.7^{+3.9}_{-7.8}$ (17.0)

**Outer Headgroup**	$35.8^{+12.7}_{-23.6}$ (46.8)	D_2O: $3.81^{+1.04}_{-1.60}$ $(4.94)^{\dagger}$<br>AuMW: $3.46^{+0.86}_{-1.27}$ $(4.34)^{\dagger}$<br>SiMW: $2.86^{+0.63}_{-0.70}$ $(3.48)^{\dagger}$<br>H_2O: $2.41^{+0.45}_{-0.27}$ $(2.82)^{\dagger}$	DPPC Headgroups: 23^{+47}_{-18} $(9)^{\dagger}$<br>LPS Headgroups: 21^{+47}_{-20} $(56)^{\dagger}$<br>Solvent: 45^{+34}_{-37} (35)	$20.7^{+3.9}_{-7.8}$ (17.0)
**Bulk Solvent**	-	D_2O: $6.19^{+0.15}_{-0.27}$ (6.31)<br>AuMW: $4.61^{+0.27}_{-0.53}$ (4.53)<br>SiMW: $1.95^{+0.64}_{-0.65}$ (1.97)<br>H_2O: $-0.11^{+0.69}_{-0.40}$ (-0.01)	Solvent: 100*	-

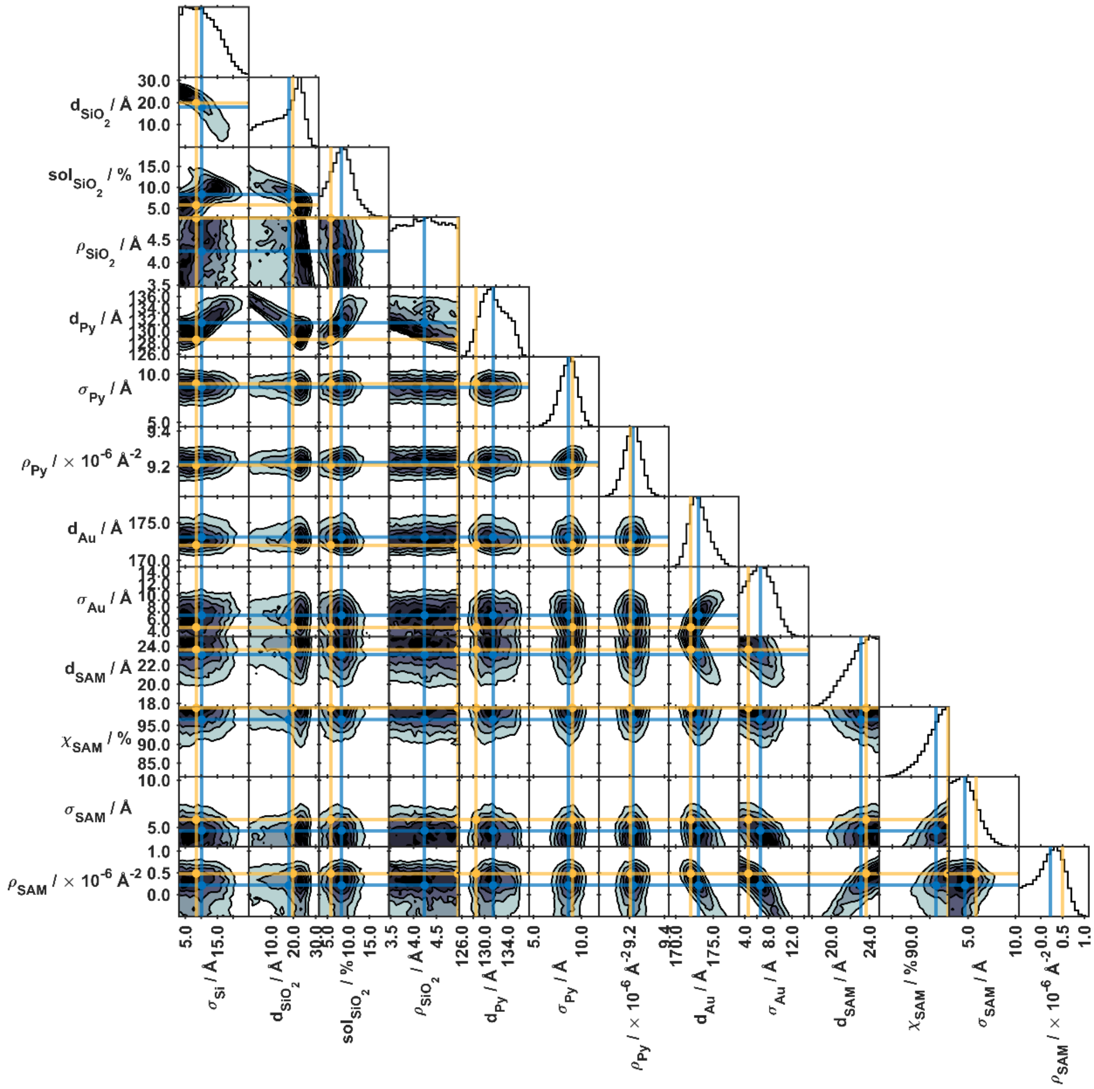

Figure S4. Corner plot corresponding to parameters describing the silicon substrate, permalloy, gold and SAM films of the sample used to investigate the interaction of AD-50 with asymmetric dDPPC/hRaLPS floating bilayers. The posterior distribution, estimated by MCMC, is shown as a histogram for each parameter on the diagonal. The contour plots represent the bivariate histograms for each parameter pair. Blue lines/points represent the median parameter value for each parameter or parameter pair, respectively. Gold lines/points represent the best-fit value for each parameter or parameter pair, respectively.

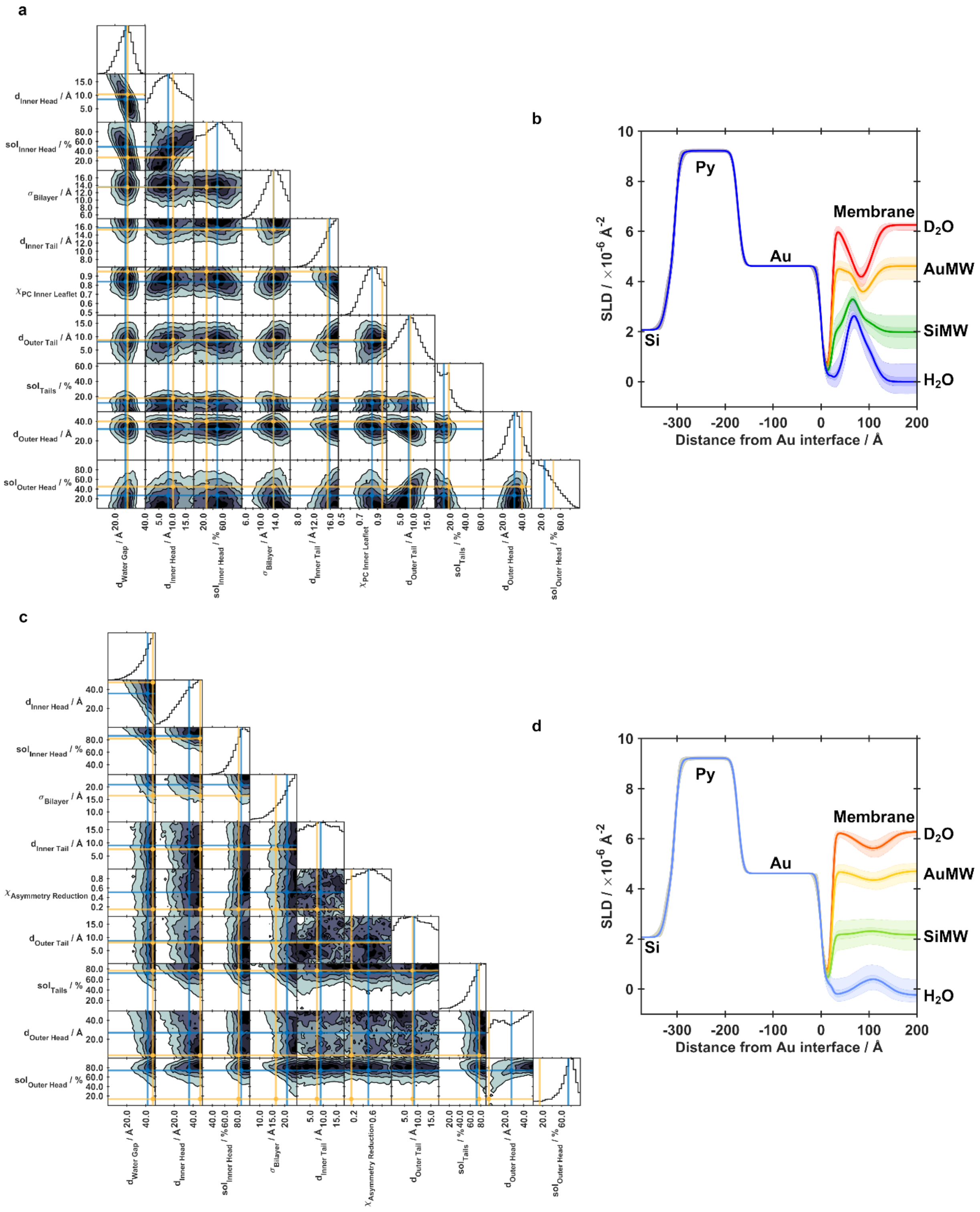

Figure S5. a) Corner plot corresponding to parameters describing the initial bilayer prior to AD-50 interaction. **b)** Complete SLD profile, generated from parameters in Figure S1 and Figure S2a, corresponding to the pristine floating asymmetric bilayer prior to AD-50 interaction. **c)** Corner plot corresponding to parameters describing the bilayer structure after AD-50 interaction. **d)** Complete SLD profile, generated from parameters in Figure S1 and Figure S2c, corresponding to the bilayer after AD-50 interaction. For corner plots, the posterior distribution, estimated by MCMC, is shown as a histogram for each parameter on the diagonal. The contour plots represent the bivariate histograms for each parameter pair. Blue lines/points represent the median parameter value for each parameter or parameter pair, respectively. Gold lines/points represent the best-fit value for each parameter or parameter pair, respectively.

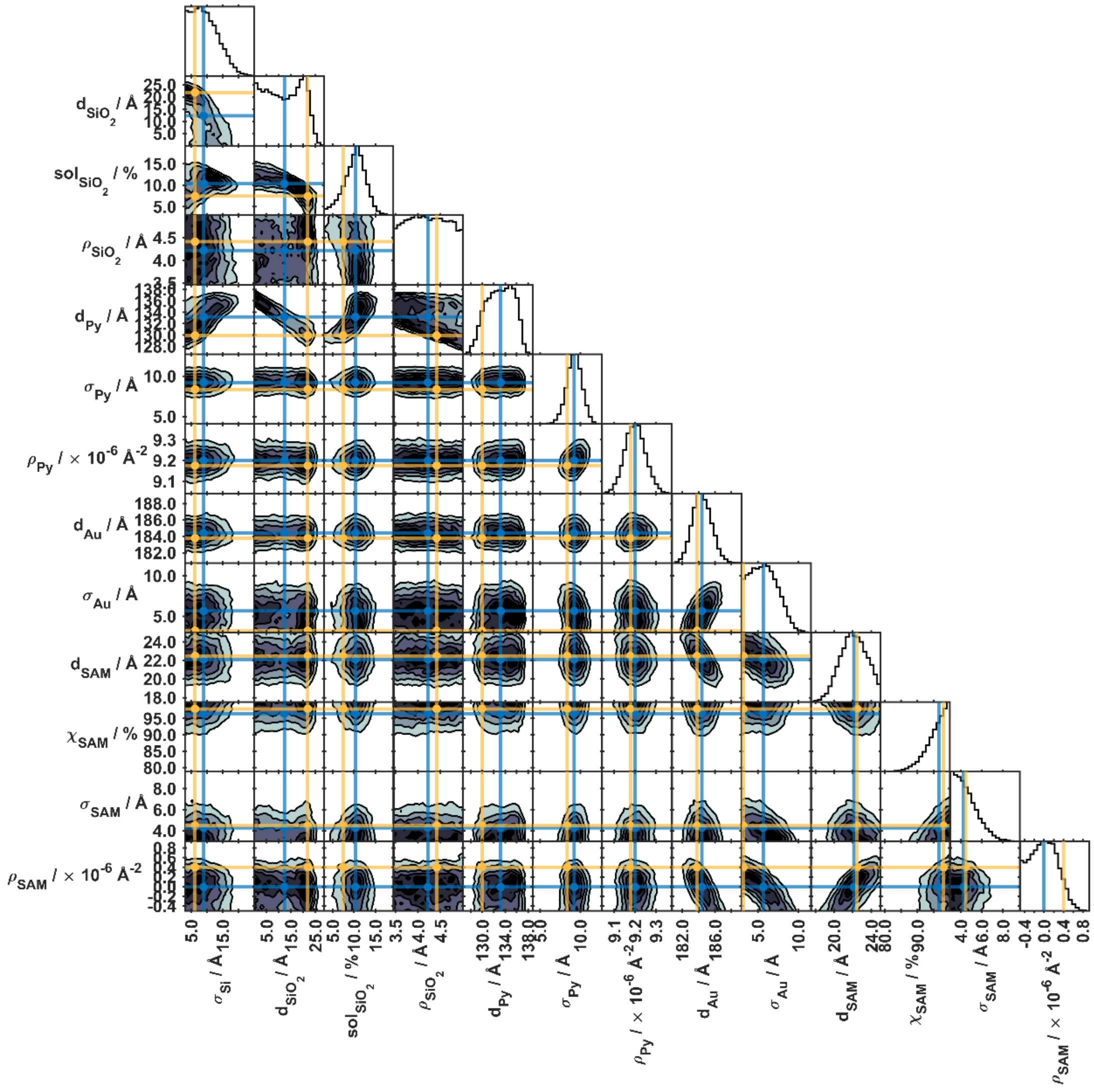

Figure S6. Corner plot corresponding to parameters describing the silicon substrate, permalloy, gold and SAM films of the sample used to investigate the interaction of AT-100-1 with asymmetric dDPPC/hRaLPS floating bilayers. The posterior distribution, estimated by MCMC, is shown as a histogram for each parameter on the diagonal. The contour plots represent the bivariate histograms for each parameter pair. Blue lines/points represent the median parameter value for each parameter or parameter pair, respectively. Gold lines/points represent the best-fit value for each parameter or parameter pair, respectively.

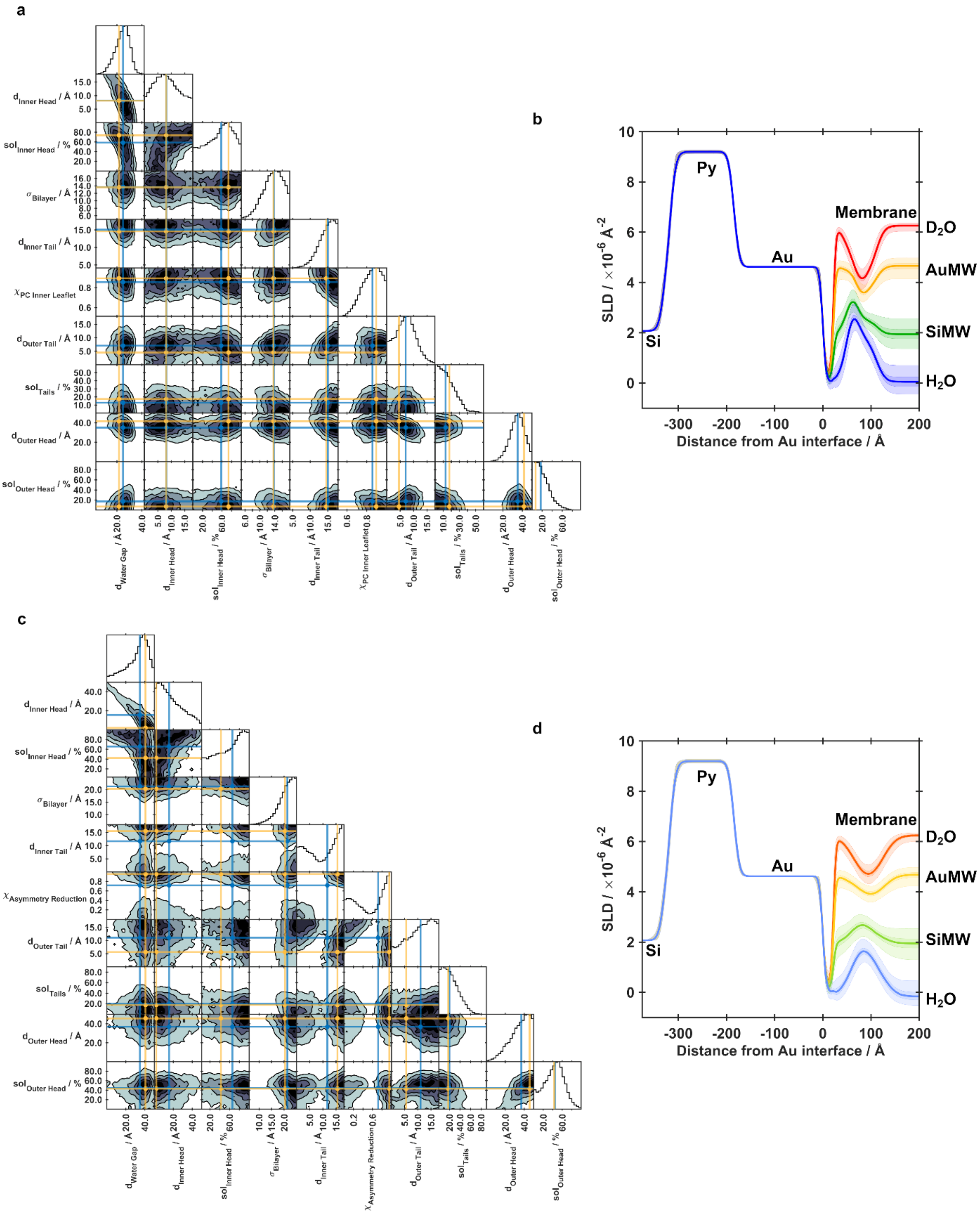

Figure S7. a) Corner plot corresponding to parameters describing the initial bilayer prior to AT-100-1 interaction. **b)** Complete SLD profile, generated from parameters in Figure S1 and Figure S2a, corresponding to the pristine floating asymmetric bilayer prior to AT-100-1 interaction. **c)** Corner plot corresponding to parameters describing the bilayer structure after AT-100-1interaction. **d)** Complete SLD profile, generated from parameters in Figure S1 and Figure S2c, corresponding to the bilayer after AT-100-1 interaction. For corner plots, the posterior distribution, estimated by MCMC, is shown as a histogram for each parameter on the diagonal. The contour plots represent the bivariate histograms for each parameter pair. Blue lines/points represent the median parameter value

for each parameter or parameter pair, respectively. Gold lines/points represent the best-fit value for each parameter or parameter pair, respectively.
